# Treadmill training improves lung function and inhibits alveolar cell apoptosis in spinal cord injured rats

**DOI:** 10.1038/s41598-024-59662-8

**Published:** 2024-04-27

**Authors:** Xianbin Wang, Yingxue Fu, Xianglian Yang, Yan Chen, Ni Zeng, Shouxing Hu, Shuai Ouyang, Xiao Pan, Shuang Wu

**Affiliations:** 1 Affiliated Hospital of Guizhou Medical University, 28 Guiyi Street, Yunyan District, Guiyang, Guizhou China; 2 Guizhou Medical University, 9 Beijing Street, Yunyan District, Guiyang, Guizhou China

**Keywords:** Spinal cord injury, Lung injury, Treadmill training, miR145-5p, MAP3K3, Medical research, Molecular medicine

## Abstract

Secondary lung injury after SCI is a major cause of patient mortality, with apoptosis playing a key role. This study aimed to explore the impact of treadmill training and miR145-5p on the MAPK/Erk signaling pathway and apoptosis in rats with complete SCI. SD rats were used to establish T10 segmental complete SCI models and underwent treadmill training 3, 7, or 14 days postinjury. Various techniques including arterial blood gas analysis, lung wet/dry weight ratio, HE staining, immunofluorescence staining, immunohistochemical staining, qRT-PCR, and Western blotting were employed to assess alterations in lung function and the expression levels of crucial apoptosis-related factors. In order to elucidate the specific mechanism, the impact of miR145-5p on the MAPK/Erk pathway and its role in apoptosis in lung cells were confirmed through miR145-5p overexpression and knockdown experiments. Following spinal cord injury (SCI), an increase in apoptosis, activation of the MAPK/Erk pathway, and impairment of lung function were observed in SCI rats. Conversely, treadmill training resulted in a reduction in alveolar cell apoptosis, suppression of the MAPK/Erk pathway, and enhancement of lung function. The gene MAP3K3 was identified as a target of miR145-5p. The influence of miR145-5p on the MAPK/Erk pathway and its impact on apoptosis in alveolar cells were confirmed through the manipulation of miR145-5p expression levels. The upregulation of miR145-5p in spinal cord injury (SCI) rats led to a reduction in MAP3K3 protein expression within lung tissues, thereby inhibiting the MAPK/Erk signaling pathway and decreasing apoptosis. Contrarily, rats with miR145-5p knockdown undergoing treadmill training exhibited an increase in miR145-5p expression levels, resulting in the inhibition of MAP3K3 protein expression in lung tissues, suppression of the MAPK/Erk pathway, and mitigation of lung cell apoptosis. Ultimately, the findings suggest that treadmill training may attenuate apoptosis in lung cells post-spinal cord injury by modulating the MAP3K3 protein through miR145-5p to regulate the MAPK/Erk signaling pathway.

## Introduction

Spinal cord injury (SCI) is a severe trauma that leads to paralysis beneath the injury segments, accompanied by severe complications, which significantly impact patients in their daily lives and societal contributions^1^. The global annual incidence of SCI is approximately 900,000 cases, with an age-standardized incidence rate of 12 per 100,000^2^. The increased mortality rate is due to secondary complications from SCI, encompassing respiratory tract and urinary tract infections, among others^3^. Respiratory complications emerge as a predominant contributor to post-SCI mortality, with varying degrees of respiratory dysfunction observed following cervical and upper thoracic SCI^4,5^. Despite this, pulmonary function tests in patients with SCI displaying lower injury segments remain suboptimal, leading to secondary lung injuries such as pulmonary hemorrhage, edema, and inflammatory infiltrates in the early stages of lower thoracic SCI with intact major respiratory muscles, suggesting that intricate mechanisms underlie SCI-induced respiratory complications^6,7^. In cervical and upper thoracic SCI, impaired respiratory muscle function and the inability to clear airway secretions contribute to respiratory dysfunction. However, the mechanisms underpinning respiratory dysfunction in lower thoracic SCI could involve apoptosis, systemic inflammatory response, and cellular autophagy^8–10^. Prior research documented the occurrence of apoptosis in lung tissues of rats following SCI, yet the precise mechanism driving apoptosis in SCI-induced lung injury remains unclear.

Exercise training is a widely employed rehabilitation strategy in clinical settings for various conditions, including neurological, musculoskeletal, and respiratory disorders. Current evidence suggests exercise training positively influences pulmonary function in patients with respiratory conditions^11^. For instance, exercise training can enhance cardiorespiratory and motor function in pulmonary fibrosis patients^12^. Animal experiments have demonstrated that aerobic exercise mitigates apoptosis in COPD mice, operating through the miR150-5p/PI3K/Akt signaling pathway to ameliorate cardiac function in chronic heart failure-afflicted rats^13,14^. However, the precise impact of treadmill training on apoptosis in lung tissues after SCI and its underlying mechanisms remain unclear.

The MAPK signaling pathway orchestrates diverse biological processes such as cell proliferation, differentiation, migration, senescence, and apoptosis, operating through various cellular mechanisms. It is widely thought that the MAPK pathway regulates apoptosis-associated occurrences, influencing the onset and progression of respiratory disorders. For instance, irisin protects against LPS-induced lung injury by inhibiting alveolar epithelial cell apoptosis^15^. Lidocaine attenuates hypoxia/reoxygenation-triggered apoptosis in lung epithelial cells by modulating the MAPK pathway^16^. However, the precise mechanism by which the MAPK signaling pathway contributes to lung cell apoptosis after SCI remains unresolved.

MicroRNA (miRNA), a highly conserved non-coding single-stranded RNA comprising approximately 22 nucleotides, has attracted substantial attention for its role in apoptosis mechanisms in lung tissues. Various studies have demonstrated miRNAs’ involvement in apoptosis mechanisms within the lungs. For instance, miR34b-5p knockdown alleviated lung cell apoptosis in LPS-induced lung injury in mice by targeting progranulin^17^. MiR-27a mitigates lung cell apoptosis and alleviates LPS-induced lung injury in mice by inhibiting the TLR4/MyD88/NF-κB signaling pathway^18^. As a member of the miRNA family, miR145-5p plays a crucial role in the pathophysiological mechanisms of diverse respiratory disorders involving apoptosis, proliferation, and differentiation. For example, Circ_YTHDF2 sponges miR145-5p to target RUNX3, ameliorating methamphetamine-induced chronic lung injury^19^. However, the effects and regulatory pathways of miR145-5p on apoptosis in rat lung tissues following SCI remain unclear.

While miR145-5p’s influence on lung injury development via apoptosis is acknowledged, its association with the MAPK signaling pathway remains unclear. Thus, this study sought to investigate whether treadmill training modulates the MAPK pathway via miR-145-5p and its relationship to apoptosis.

## Material and methods

### Animals

Adult female specific pathogen-free (SPF) Sprague–Dawley (SD) rats (body weight: 220–250 g; age: 8–10 weeks) were purchased from Liaoning Changsheng Biotechnology Co., Ltd, Certificate of Conformity No.: SCXK(Liao)2020–0001. The animals were housed under controlled conditions, including a room temperature of 22–26 °C, relative humidity of 40–60%, and a day/night cycle of 12/12 h, and were provided water and food ad libitum. After 7 days of acclimatization feeding, the rats were divided into the following groups: Sham group (n = 45), SCI group (n = 45), SCI exercise group (SCI+TT group, n = 45), normal control group (control group, n = 6), non-load group (n = 6), negative control+SCI group (NC+SCI group, n = 30), NC+SCI+TT group (n = 15), AAV9-miR145-5p group (n = 6), AAV9-miR145-5p/SCI group (n = 15), AAV9-miR145-5p inhibition group (n = 6), AAV9-miR145-5p inhibition /SCI group (n = 15) and AAV9-miR145-5p inhibition SCI+TT group (n = 15) by using the random number table method. The protocols related to animal experiments were approved by the Ethics Committee for the Use of Animals of Guizhou Medical University, license No. 2000949.

### Establishment of the SCI rat model

After intraperitoneal anesthesia with 3% pentobarbital sodium (30 mg/kg), the rats were fixed prone on the operating table, and the skin was prepared and disinfected in the routine operation area. The skin, fascia, and paravertebral muscles of the rat’s back were incised longitudinally layer by layer to expose the vertebral arch roots of the spinal segments from T9 to T11, and the arch roots on both sides of T10 were cut off with bone-biting forceps^20^. Subsequently, the spinal cord was fully severed at the T10 spinal cord segment. At the point of severance, the rat’s tail exhibited swinging or bending motion, followed by hind limb paralysis following convulsions. The 2-mm-wide spinal cord tissue was resected. Two researchers confirmed that the spinal cord was completely transected under direct visualization. Hemostatic sponges were used to stop bleeding locally and were then sutured layer by layer. In the sham-operated group, only the spinal cord was exposed without dissecting it. After surgery, penicillin was injected intraperitoneally for 3 consecutive days, and the lower abdomen of the rats was massaged to assist urination four times per day.

The Basso, Beattie, and Bresnahan (BBB) score assessment was performed after the rats were awake from anesthesia after surgery, and the final score for each rat was determined by averaging the scores of the two observers. Scores ranged from 0 to 21 (with 0 indicating no locomotion and 21 indicating normal locomotion)^21^. A score of 0 for both lower limbs on the day of surgery was considered successful modeling.

### Treadmill training

The rats in the SCI exercise group (SCI+TT group) began exercise training three days after the operation and the rats underwent treadmill training on a small animal treadmill (model: ZS-PT, Zhongshi Technology, Beijing, China) twice a day (one cycle of continuous exercise for 10 min with 5 min of rest, and three cycles of one exercise training session at a speed of 5 m/min)^22^.

### Measurement of pulmonary edema

The procedures for measurement of pulmonary edema were performed as previously reported^23^. Rats were anesthetized, the thoracic cavity was opened, and intact lung lobes on both sides were removed to evaluate pulmonary edema. Lung tissues were cleared of extrapulmonary tissues, weighed immediately to obtain wet weights, and then dried at 60 °C for 48 h so that the tissues were kept at a constant weight and weighed again to obtain dry weights. The wet weight/dry weight ratio was calculated to analyze the degree of pulmonary edema.

### Blood gas analysis

The procedures for blood gas analysis were performed as previously reported^24^. Rats were anesthetized, and arterial blood samples were drawn via the abdominal aorta with a blood needle on days 3, 7, and 14, respectively. The pH, partial pressure of oxygen (PaO_2_), and partial pressure of carbon dioxide (PaCO_2_) in the samples were measured immediately using a blood gas analyzer (Nova Biochemical, Waltham, US).

### Hematoxylin–eosin (HE) staining

The procedures for HE staining were performed as previously reported^25^. Rats were anesthetized, and the lung lobes were taken after transcardiac perfusion with cold saline and 4% paraformaldehyde, fixed in paraformaldehyde at room temperature, dehydrated by gradient alcohol, embedded in paraffin, and stored at room temperature. Paraffin Sects. (4µm thick) were deparaffinized and stained with HE. Lung pathophysiologic changes were observed with an ordinary light microscope and scored for lung injury pathology at high magnification (400 × magnification), with the pathologist blinded to the grouping. Histological scoring was based on inflammatory cell infiltration, edema, congestion, and intra-alveolar hemorrhage (0 as normal, 1 as mild, 2 as moderate, and 3 as severe), with a maximum score of 12.

### Immunohistochemistry staining

The procedures for immunohistochemistry staining were performed as previously reported^26^. Paraffin Sects. (4µm thick) were deparaffinized, hydrated, and repaired with EDTA antigen, blocked with 15% hydrogen peroxide to block endogenous peroxidase, and closed with a 10% goat serum solution. They were incubated with PBS-diluted Erk (1:500, Proteintech, Wuhan, China, Cat No. 51068–1-AP), caspase 9 (1:500, Proteintech, Wuhan, China, Cat No.66169–1-Ig), and caspase 3 (1:500, Proteintech, Wuhan, China, Cat No.19677–1-AP), respectively, overnight at 4 °C. After incubation at 4 °C overnight, the slices were combined with secondary antibodies (1:500, Abcam, Cambridge, UK) and then stained with DAB. The staining was terminated when positive expression was observed under a light microscope, and the slices were re-stained with hematoxylin, then dehydrated step by step after differentiation, and then blocked with a neutral resin. The positive rate was calculated by the positive coloring cell counting method, positive rate = (number of positive cells/total number of cells) × 100%.

### Immunofluorescence staining

The procedures for immunofluorescence staining were performed as previously reported^27^. Paraffin Sects. (4 μm thick) were deparaffinized, and the sections were antigenically repaired with 0.01 M citrate buffer. Sections were labeled with antibodies Bax (1:400, Proteintech, Wuhan, China, Cat No.60267-1-Ig), Bcl-2 (1:400, Proteintech, Wuhan, China, Cat No.60178-1-Ig), and CytC (1:400, Proteintech, Wuhan, China, Cat No.10993-1-AP), and then incubated with the appropriate secondary antibodies (1:300, Proteintech, Wuhan, China), the nuclei were identified by DAPI (1:100, Abcam, Cambridge, UK) staining, and the positive staining was indicated by a fluorescent green dot. Positive rate = (number of positive cells/total number of cells) × 100%.

For the TUNEL assay^28^, after routine dehydration, hydration, and repair of paraffin sections of each set of brain tissues, 100 µl of proteinase K (20 µg/ml) was added to each sample according to the instructions of the TUNEL kit and incubated for 30 min at room temperature. Then, 50 µl of TUNEL assay was added dropwise to the samples and incubated for 60 min at 37 °C, protected from light. Finally, DAPI (1:100, Abcam) was added, and incubated for 5 min at room temperature away from light, washed three times with PBS, and sealed with a drop of anti-fluorescent extractant. At the end of it, the sections were observed under an orthogonal fluorescence microscope.

### Western blotting

The procedures for western blotting were performed as previously reported^29^. Rats were anesthetized, and the lung tissues were dissected and removed by cardiac perfusion with cold saline and frozen at − 80 °C for storage. After ultrasonic crushing of the tissues, proteins were extracted with RIPA lysate (Solarbio, Beijing, China) mixed with PMSF (100:1). After detecting the protein concentration according to the instructions of the BCA kit (Solarbio, Beijing, China), adding loading buffer for high-temperature denaturation, and using SDS-PAGE gel electrophoresis, 30µg of protein per well was loaded, transferred to the PVDF membrane, and blocked with 5% skimmed milk. The blots were cut prior to hybridisation with antibodies for convenience. Then they were incubated at 4 °C overnight with MAP3K3 (1:1000, Proteintech, Wuhan, China, Cat No. 21072-1-AP), Erk (1:1000, Proteintech, Wuhan, China, Cat No. 51068-1-AP), caspase 9 (1:1000, Proteintech, Wuhan, China, Cat No. 66169-1-Ig), caspase 3 (1:1000, Proteintech, Wuhan, China, Cat No. 19677-1-AP), Bax (1:1000, Proteintech, Wuhan, China, Cat No. 60267-1-Ig), Bcl-2 (1:1000, Proteintech, Wuhan, China, Cat No. 60178-1-Ig), CytC (1:1000, Proteintech, Wuhan, China, Cat No. 10993-1-AP) primary antibodies. When washed with TBST, it was then combined with horseradish peroxidase (HRP)-coupled secondary antibody (1:5000, Proteintech, Wuhan, China), visualized by ECL analysis (Millipore, US), and used as a reference for quantification by glyceraldehyde 3-phosphate dehydrogenase (GAPDH, Proteintech, Wuhan, China, Cat No. 10494-1-AP).

### qRT-PCR

The procedures for qRT-PCR were performed as previously reported^30^. Lung tissues cryopreserved at − 80 °C were removed, ruptured by ultrasound and homogenized with 1 ml of Trizol reagent (Ambion) to extract total RNA. First-strand cDNA was synthesized using the RT kit (Takara) and the miRNA First-strand cDNA Synthesis kit (Stem Loop Method) according to the manufacturer’s instructions. Real-time PCR was performed using the TB-Green RT-PCR kit on the 7500 Real-Time PCR System (Applied Biosystems) using the TB-Green RT-PCR kit, and the relative expression levels of the target genes were calculated by the 2^−ΔΔCT^ method, with GAPDH and U6 as internal references. The primer sequences are shown in Table 1.

**Table 1:** The primer sequences.

Gene name	Forward primer (5′-3′)	Reverse primer(5′-3′)
miR145-5p	CGGTCCAGTTTTCCCAGGA	AGTGCAGGGTCCGAGGTATT
Bax	CAGGATCGAGCAGAGAGGA	TGTCCAGTTCATCGCCAA
Bcl-2	GGG GAAACAC C AG AATC A	GGCCCGAAAGAGAGAAA
CytC	CTGGATTCTCTTACACAGATGCC	TGCCCTTTCCTTCTTCTTAATTCC
Caspase3	GCGGTATTGAGACAGACAGTGGAAC	AACCATGACCCGTCCCTTGAATTTC
Caspase 9	ATCCTTGTGTCCTACTCCACCTTCC	CAGCATTGGCGACCCTGAGAAG
U6	AGAGAAGATTAGCATGGCCCCTG	ATCCAGTGCAGGGTCCGAGG
GAPDH	AGGTCGGTGTGAACGGATTTG	TGTAGACCATGTAGTTGAGGTCA

### Dual-luciferase reporter gene assay

The procedures for dual-luciferase reporter gene assay were performed as previously reported^31^. Wild-type and mutant binding site sequences for MAP3K3 were designed based on predictions found in the miRWalk, miRDB, and ENCORI databases. The binding site sequences were cloned into the pmirGLO vector. The pGL3-MAP3K3-WT vector, the pGL3-MAP3K3-Mut vector, miR145-5p MIMIC, and MAP3K3-NEG were randomly introduced under four different transfection conditions (MIC-NEG+MT2-WT group, MIC-NEG+MT2-Mut group, MIC+MT2-WT group, and MIC+MT2-Mut group) for 48 h and were analyzed with a Thermo Fisher Science chemiluminescence analyzer to detect luciferase activity.

### Overexpression and knockdown of miR145-5p in the lungs of rats

MiR145-5p was either overexpressed or knocked down using an adeno-associated virus (AAV) from Jikai, China. The AAV was diluted in 200 µL of PBS. Rats were anesthetized and secured on a small animal tracheal intubation platform. The rat laryngoscope was used to expose the vocal fissure. The nozzle of an intrapulmonary nebulizer drug delivery device (Yuande Kaisen, China) was inserted into the trachea, and then it was retracted 1–2 mm after reaching the rongeur before being firmly advanced into AAV suspension at once^32^.

### Statistical analysis

Statistical tests were performed using SPSS software (version 23.0; SPSS, Chicago, IL, USA) and GraphPad Prism 9.0 (GraphPad Software Inc., San Diego, CA, USA) using one-way or two-way analysis of variance (ANOVA) for multiple comparisons, followed by Tukey’s test. Statistical significance was set at *P* < 0.05.

## Results

### Treadmill training improves pulmonary dysfunction after SCI

**Figure 1:**
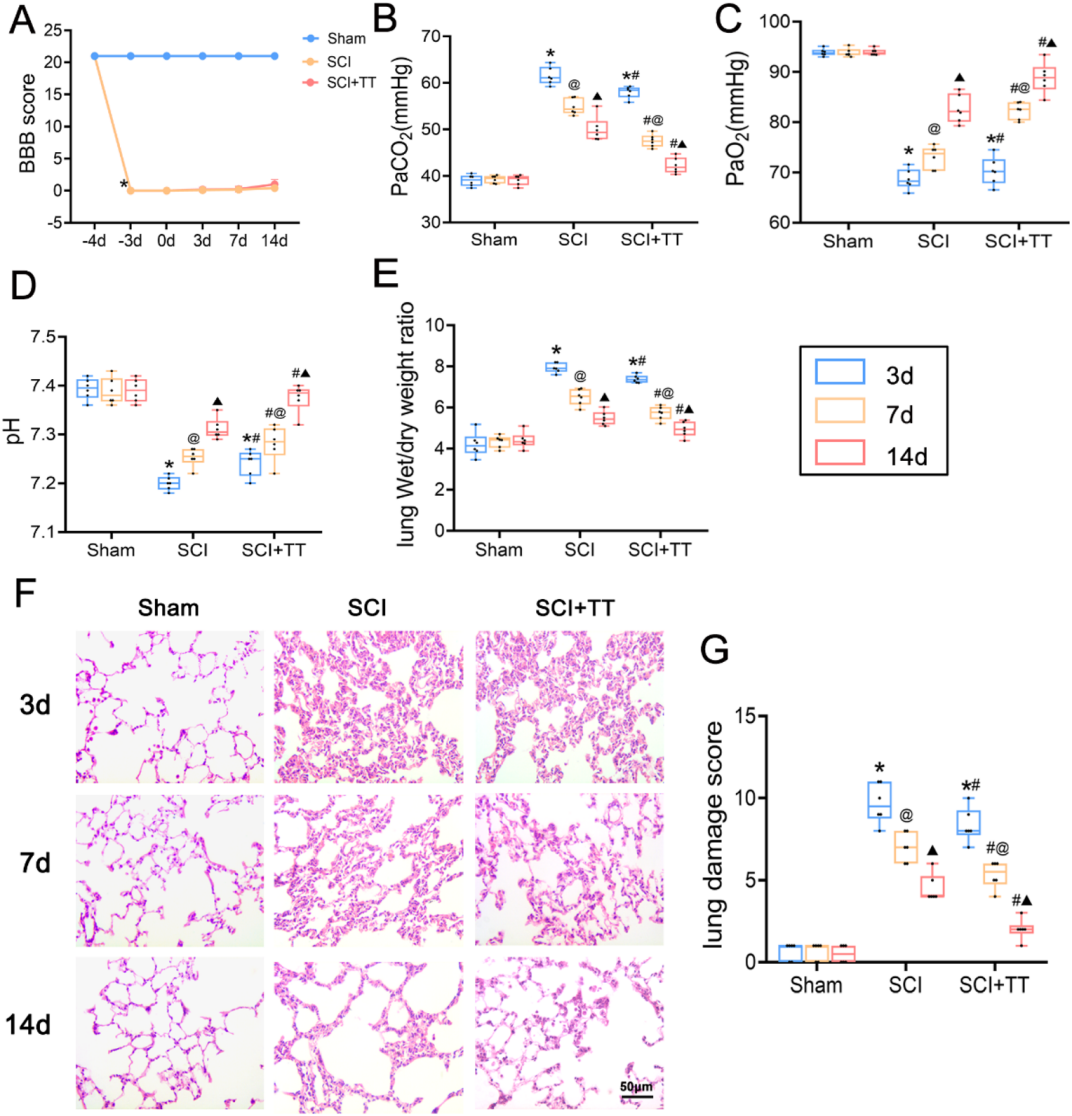
Effects of treadmill training on lung function and lung histopathologic morphology in SCI rats. (**A**) BBB scores, n = 15. (**B**) PaCO_2_ level, n = 6. (**C**) PaO_2_ level, n = 6. (**D**) pH level, n = 6. (**E**) Lung wet/dry weight ratio, n = 6. (**F**) Lung tissues HE staining results, n = 6, scale bar = 50 µm. (**G**) Lung pathology score results. *P < 0.05, compared with the Sham group at the same time point; #*P* < 0.05, compared with the SCI group at the same time point; @*P* < 0.05, compared with the same group at day 3; ▲*P* < 0.05, compared with the same group at day 7. *P* < 0.05 was determined by two-way ANOVA (Bonferroni’s multiple comparison test). PaO_2_: partial pressure of oxygen; PaCO_2_. carbon dioxide partial pressure.

To determine the successful establishment of the SCI rat model, the BBB score assessment was employed (Fig. 1A). Rats in the Sham group achieved a score of 21, while postoperative SCI and SCI+TT groups scored 0, confirming the successful establishment of complete SCI at the T10 segment. To assess the impact of SCI and treadmill training on rat pulmonary function, PaO_2_, PaCO_2_, and pH in the abdominal aorta were measured during arterial blood gas analysis (Fig. 1B–D). Results demonstrated that compared to the Sham group, SCI induced reduced pH and PaO_2_ levels alongside increased PaCO_2_ levels (*p* < 0.05). Conversely, the SCI+TT group exhibited elevated pH and PaO_2_ along with reduced PaCO_2_ compared to the SCI group (*p* < 0.05). Lung edema was assessed using wet/dry weight ratios (Fig. 1E), revealing significant edema in the SCI and SCI+TT groups compared to the Sham group (*p* < 0.05). Notably, the wet/dry weight ratio was significantly lower in the SCI+TT group compared to the SCI group (*p* < 0.05). Pathological examination using HE staining (Fig. 1F–G) revealed inflammatory cell infiltration, edema, congestion, and intra-alveolar hemorrhage in SCI-afflicted rat lung tissues. However, the pathological score attributed to lung injury was notably lower in the SCI+TT group versus the SCI group (*p* < 0.05). These findings substantiated an improvement in lung function and histopathological changes over time post-SCI. Treadmill training significantly mitigated lung tissue damage and pulmonary dysfunction, leading to a shortened duration of lung injury after SCI.

These results collectively suggest that SCI triggered lung tissue damage, resulting in pulmonary dysfunction, whereas treadmill training attenuated pathological lung tissue damage, consequently alleviating pulmonary dysfunction.

### Apoptosis increases in rat lung tissues after SCI and treadmill training inhibits apoptosis

**Figure 2:**
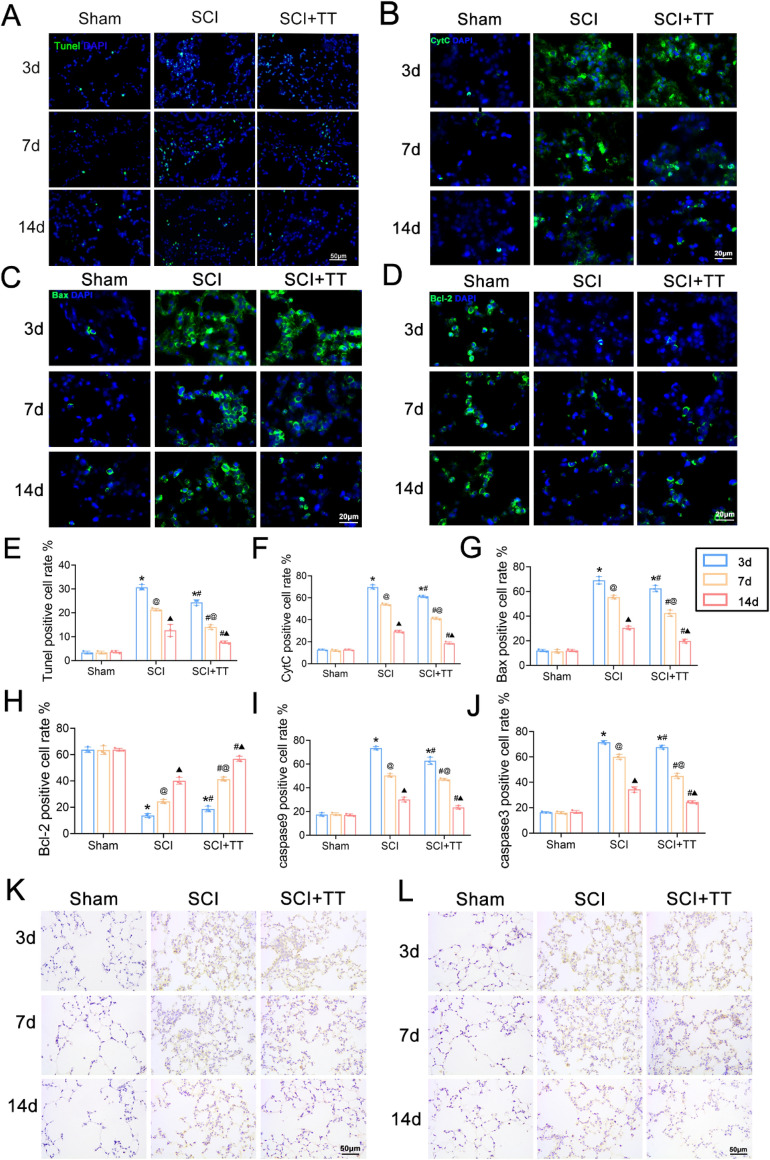
Effects of treadmill training on lung cell apoptosis in rats following SCI. (**A**) TUNEL staining, scale bar = 50 µm. (**B**) CytC immunofluorescence staining, scale bar = 20 µm. (**C**) Bax immunofluorescence staining, scale bar = 20 µm. (**D**) Bcl-2 immunofluorescence staining, scale bar = 20 µm. (**E**) TUNEL staining-positive cell rate, n = 3. (**F**) CytC-positive cell rate, n = 3. (**G**) Bax-positive cell rate, n = 3. (**H**) Bcl-2-positive cell rate, n = 3. (**I**) caspase 9-positive cell rate, n = 3. (**J**) caspase 3-positive cell rate, n = 3. (**K**) Immunohistochemical staining for caspase 9, scale bar = 50 µm. **(**L) caspase 3 immunohistochemical staining, scale bar = 50 µm. *P < 0.05, compared with the Sham group at the same time point; #*P* < 0.05, compared with the SCI group at the same time point; @*P* < 0.05, compared with the same group on day 3; ▲*P* < 0.05, compared with the same group on day 7.* P* < 0.05 was determined by two-way ANOVA (Bonferroni’s multiple comparison test).

**Figure 3:**
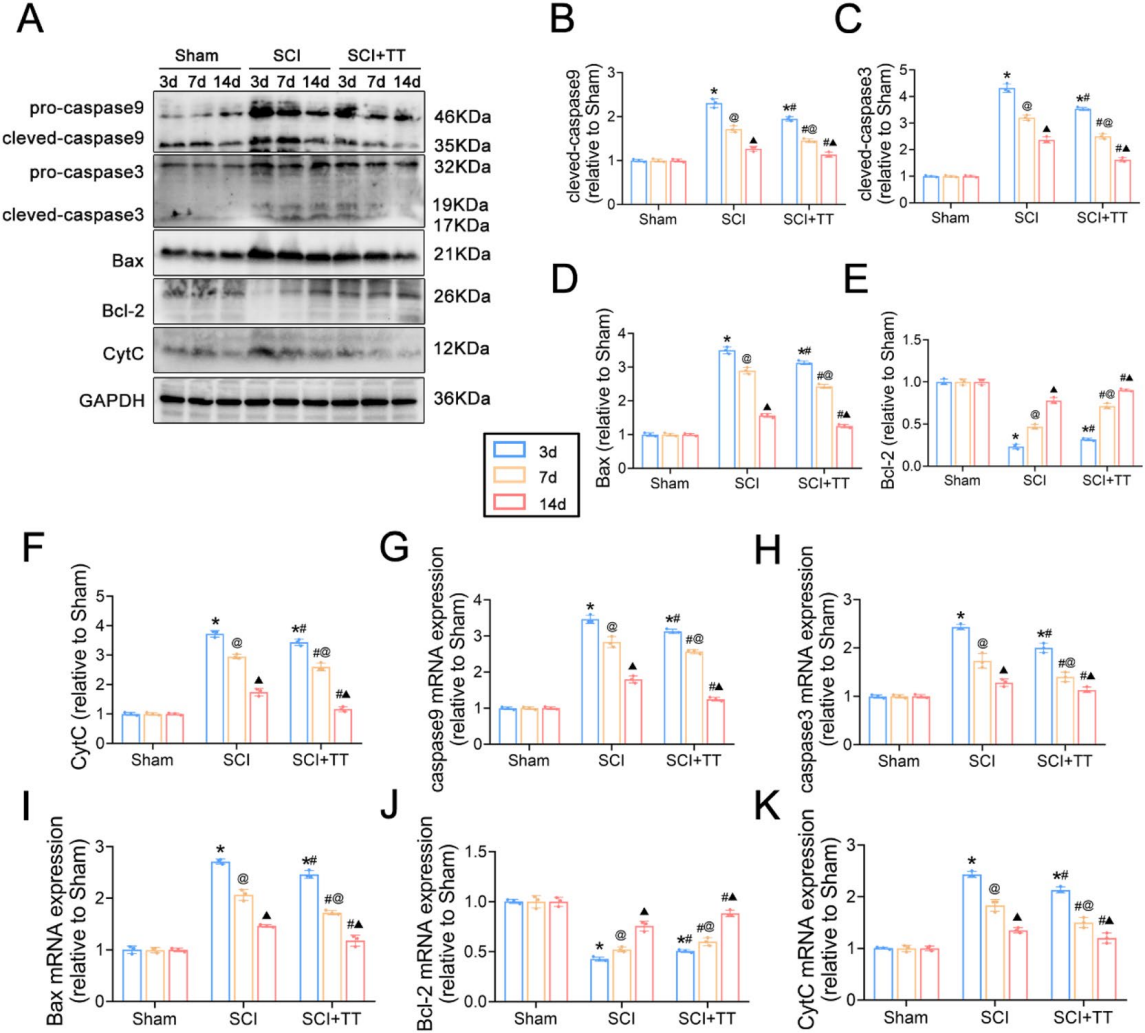
(**A**) Western blotting detection of caspase 9, caspase 3, Bax, Bcl-2, and CytC expression. (**B**) Relative expression levels of caspase 9. (**C**) Relative expression levels of caspase 3. (**D**) Relative expression levels of Bax. (**E**) Relative expression levels of Bcl-2. (**F**) Relative expression levels of CytC. (**G**) Relative expression levels of caspase 9 mRNA. (**H**) Relative expression levels of caspase 3 mRNA. (**I**) Relative expression levels of Bax mRNA. (J) Relative expression levels of Bcl-2 mRNA. (**K**) Relative expression levels of CytC mRNA.*P < 0.05, compared with the Sham group at the same time point; #*P* < 0.05, compared with the SCI group at the same time point; @*P* < 0.05, compared with the same group on day 3; ▲*P* < 0.05, compared with the same group on day 7. *P* < 0.05 was determined by two-way ANOVA (Bonferroni’s multiple comparison test).

TUNEL staining (Fig. 2A,E) demonstrated a significant increase in apoptotic cells in the SCI group compared to the Sham group, which significantly decreased following treadmill training (*P* < 0.05). Immunohistochemical staining results (Fig. 2I–L) indicated significantly higher expression levels of caspase 9 and caspase 3 in the SCI group compared to the Sham group, with significantly lower levels observed in the SCI+TT group compared to the SCI group (*P* < 0.05). Immunofluorescence staining (Fig. 2B–D,F–H) revealed that Bax and CytC expression levels increased in the SCI group compared to the Sham group, while Bcl-2 expression levels decreased (*P* < 0.05). Conversely, treadmill training led to decreased Bax and CytC expression levels and increased Bcl-2 expression levels (*P* < 0.05). Western blotting and qRT-PCR (Fig. 3A–K) revealed consistent results, illustrating elevated expression levels of caspase 9, caspase 3, Bax, and CytC, coupled with reduced expression Bcl-2 levels in the SCI group versus the Sham group (*P* < 0.05). On the other hand, the SCI+TT group displayed significantly elevated Bcl-2 expression levels and reduced caspase 9, caspase 3, Bax, and CytC expression levels compared to the SCI group (*P* < 0.05).

Taken together, these findings indicate the activation of apoptotic mechanisms in lung tissues following SCI, which was effectively mitigated by treadmill training.

### The MAPK signaling pathway is activated in lung tissue after SCI and inhibited by treadmill training

**Figure 4:**
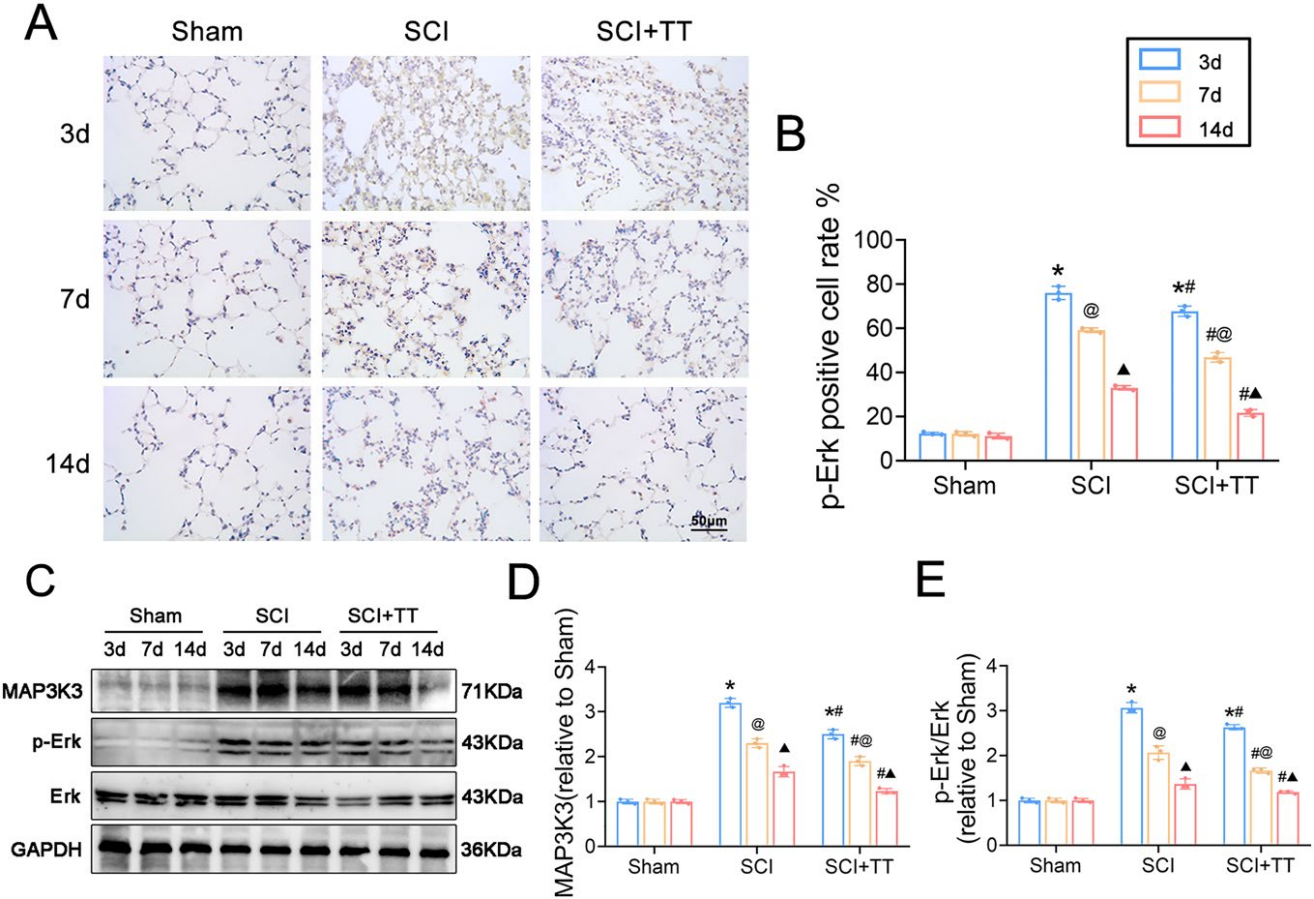
Effects of treadmill training on the MAPK signaling pathway in lung tissue of rats with SCI. (**A**) Immunohistochemical staining of p-Erk, scale bar = 50 µm. (**B**) Rate of p-Erk positive cells, n = 3. (**C**) Western blotting detection of MAP3K3 and p-Erk protein expression. (**D**) MAP3K3 protein relative expression levels. (E) Relative protein expression levels of p-Erk. *P < 0.05, compared with the Sham group at the same time point; #*P* < 0.05, compared with the SCI group at the same time point; @*P* < 0.05, compared with the same group on day 3; ▲*P* < 0.05, compared with the same group on day 7. *P* < 0.05 was determined by two-way ANOVA (Bonferroni’s multiple comparison test).

Immunohistochemical staining (Fig. 4A–B) demonstrated that p-Erk expression in the SCI group was significantly higher than in the Sham group, while the SCI+TT group exhibited reduced p-Erk expression compared to the SCI group (*P* < 0.05). Western blotting results (Fig. 4C–E) yielded consistent findings, showing significantly higher relative expression levels of MAP3K3 and p-Erk protein in the SCI group compared to the Sham group, with significantly lower levels observed in the SCI+TT group compared to the SCI group (*P* < 0.05).

These findings collectively suggest that the MAPK signaling pathway becomes activated in lung tissues after SCI and treadmill training effectively suppresses this activation.

### MAP3K3 is a target gene of miR145-5p, and overexpression of miR145-5p attenuates lung injury after SCI in rats

**Figure 5:**
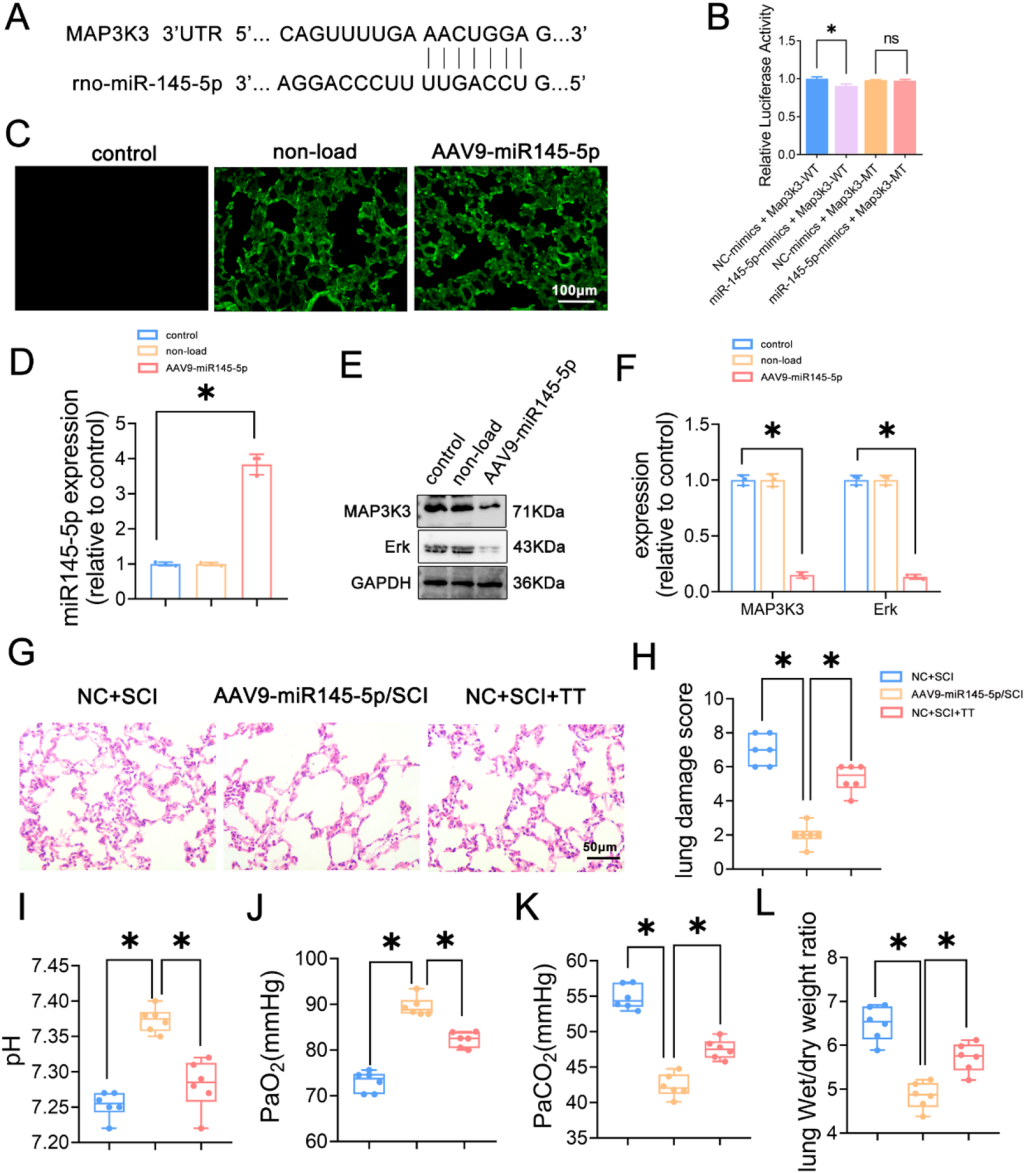
MAPK is a target gene of miR145-5p, and the effects of miR-145-5p overexpression and treadmill training on lung function in SCI rats. (**A**) Predicted sequence of miR145-5p and binding site of MAP3K3. (**B**) Dual luciferase reporter gene assay. (**C**) EGFP fluorescent labeling assay, scale bar = 100 µm. (**D**) miR145-5p relative expression levels. (**E**) Western blotting detection of MAP3K3, Erk. (**F**) Relative protein expression levels of MAP3K3, Erk. (**G**) HE staining results of Lung tissues, scale bar = 50 µm. (**H**) Lung pathology scoring results, n = 6. (**I**) pH level, n = 6. (**J**) PaO_2_ level, n = 6. (K) PaCO_2_ level, n = 6. (**L**) Lung wet/dry weight ratio, n = 6. *P < 0.05 as determined by one-way ANOVA (Tukey’s multiple comparison test).

To explore the role of miR145-5p in lung injury after SCI and its underlying mechanisms, bioinformatics databases (TargetScan, miRDB) were utilized to predict the sequence of miR145-5p and its binding site on MAP3K3 (Fig. 5A). This regulatory relationship was verified using dual luciferase reporter gene assay (Fig. 5B), substantiating that co-transfection of miR145-5p mimic and MAP3K3 3ʹUTR WT significantly reduced luciferase expression compared to the mimic-NC group (*P* < 0.05). However, there was no significant difference in luciferase expression in the MAP3K3 3ʹUTR MUT group (*P* > 0.05), indicating miR145-5p’s targeting of the MAP3K3 gene.

In addition, examination of plasmid EGFP fluorescence labeling through fluorescence microscopy displayed fluorescence in both non-loaded viral and overexpression groups’ lung tissues, whereas no fluorescence was observed in the saline control group (Fig. 5C).

To substantiate successful miR145-5p overexpression and its influence on the MAPK signaling pathway, qRT-PCR and Western blotting were employed to assess miR145-5p expression and the MAPK signaling pathway (Fig. 5D–F). Compared to the control group, the non-loaded group exhibited no significant alterations in miR145-5p expression levels, MAP3K3, and Erk protein expression levels (*P* > 0.05). However, the overexpression group displayed significantly upregulated miR145-5p expression levels alongside diminished MAP3K3 and Erk protein levels (*P* < 0.05).

Next, rats underwent transfection with an empty carrier virus and an overexpressed miR145-5p AAV for 14 days to establish SCI modeling, followed by lung function assessment on the 7th day (Fig. 5I–K). Findings revealed that the AAV9-miR145-5p/SCI group exhibited significantly elevated pH and PaO_2_ levels and notably reduced PaCO_2_ levels in comparison to both the NC+SCI and the NC+SCI+TT groups (*p* < 0.05). Additionally, the assessment of lung edema using the wet/dry weight ratio was conducted across all groups (Fig. 5L), with results indicating a significantly lower ratio in the AAV9-miR145-5p/SCI group relative to the NC+SCI group and the NC+SCI+TT group (*p* < 0.05). Further observation of lung tissue pathological changes was performed through HE staining, coupled with pathological scoring (Fig. 5G–H). Clear signs of inflammatory cell infiltration, edema, congestion, and intra-alveolar hemorrhage were observed in the lung tissues of both the NC+SCI group and the NC+SCI+TT group. However, the AAV9-miR145-5p/SCI group exhibited notably reduced pathological scores of lung injury compared to the other two groups (*p* < 0.05).

Taken together, these results suggest the role of MAP3K3 as a miR145-5p target gene. Furthermore, overexpression of miR145-5p effectively mitigated lung injury post-SCI in rats. Notably, direct miR145-5p overexpression exhibited a greater reduction in lung injury in SCI rats compared to treadmill training.

### Overexpression of miR145-5p inhibits the MAPK signaling pathway and suppresses apoptosis in rat lung tissues after SCI

**Figure 6:**
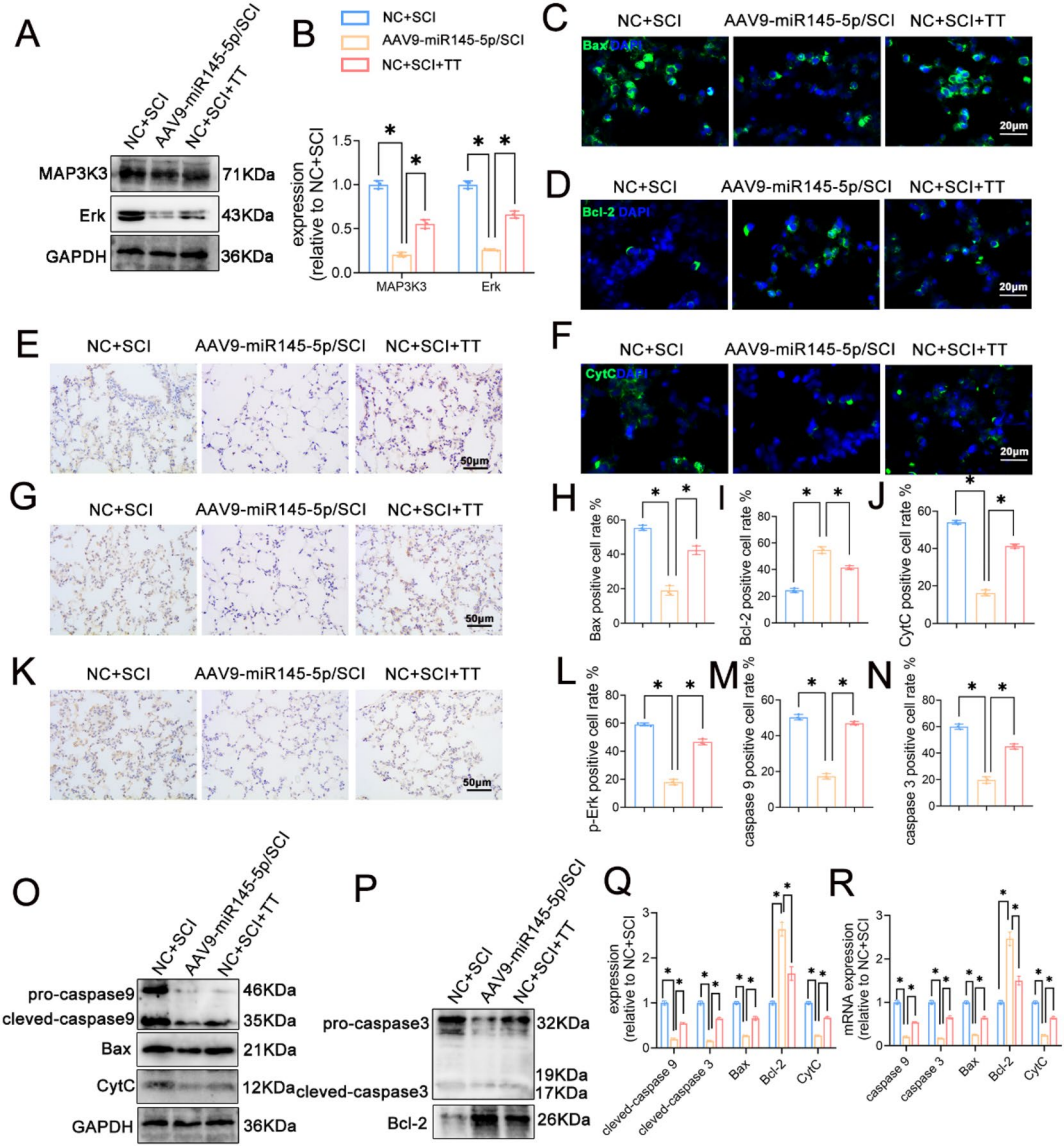
Effect of overexpression of miR145-5p on the MAPK signaling pathway and lung cell apoptosis in rat lung tissues post-SCI. (**A**) Western blotting detection of MAP3K3, Erk expression. (**B**) Relative protein expression levels of MAP3K3, Erk. (**C**) Bax immunofluorescence staining, scale bar = 20 µm. (**D**) Bcl-2 immunofluorescence staining, scale bar = 20 µm. (**E**) p-Erk immunohistochemical staining, scale bar 50 = µm. (**F**) CytC immunofluorescence staining, scale bar = 20 µm. (**G**) caspase 9 immunohistochemical staining, scale bar = 50 µm. (**H**) Bax-positive cell rate, n = 3. (**I**) Bcl-2-positive cell rate, n = 3. (**J**) CytC-positive cell rate, n = 3. (**K**) caspase 3 immunohistochemical staining, scale bar 50 = µm. (**L**) p-Erk-positive cell rate, n = 3. (**M**) caspase 9-positive cell rate, n = 3. (**N**) caspase 3-positive cell rate, n = 3. (**O**–**P**) Western blotting detection of caspase 9, Bax, Bcl2, and CytC expression. (**Q**) Relative protein expression levels of caspase 9, caspase 3, Bax, Bcl-2, and CytC. (**R**) Relative expression levels of caspase 9, caspase 3, Bax, Bcl-2, CytC mRNA. *P < 0.05 as determined by one-way ANOVA (Tukey’s multiple comparison test).

Immunohistochemical staining results (Fig. 6E,L) revealed significantly reduced p-Erk expression in the AVV9-miR145-5p/SCI group in comparison to both the NC+SCI group and the NC+SCI+TT group (*P* < 0.05). Correspondingly, Western blotting outcomes (Fig. 6A–B) demonstrated significantly lower expression levels of MAP3K3 and Erk protein in the AVV9-miR145-5p/SCI group relative to the NC+SCI group and the NC+SCI+TT group (*P* < 0.05).

Lung cell apoptosis was further assessed, with immunohistochemical staining (Fig. 6G,K,M,N) revealing notably reduced expression of caspase 9 and caspase 3 in the AVV9-miR145-5p/SCI group compared to both the NC+SCI group and the NC+SCI+TT group (*P* < 0.05). Immunofluorescence staining (Fig. 6C–D,F,H–J) consistently indicated the decreased abundance of Bax and CytC positive cells, along with increased Bcl-2 expression in the AVV9-miR145-5p/SCI group relative to the other two groups (*P* < 0.05). Additionally, Western blotting and qRT-PCR (Fig. 6O–R) further supported these findings, demonstrating significantly higher expression levels of Bcl-2 protein and mRNA, while expressions levels of caspase 9, caspase 3, Bax, and CytC protein and mRNA were significantly lower in the AVV9-miR145-5p/SCI group compared to the other two groups (*P* < 0.05).

Taken together, these results collectively indicate that miR145-5p overexpression effectively suppresses the MAPK signaling pathway and mitigates lung cell apoptosis in rat lung tissues following SCI.

### Knockdown of miR145-5p increases MAPK expression and aggravates lung injury after SCI in rats, and treadmill training attenuates lung injury in knockdown rats

**Figure 7:**
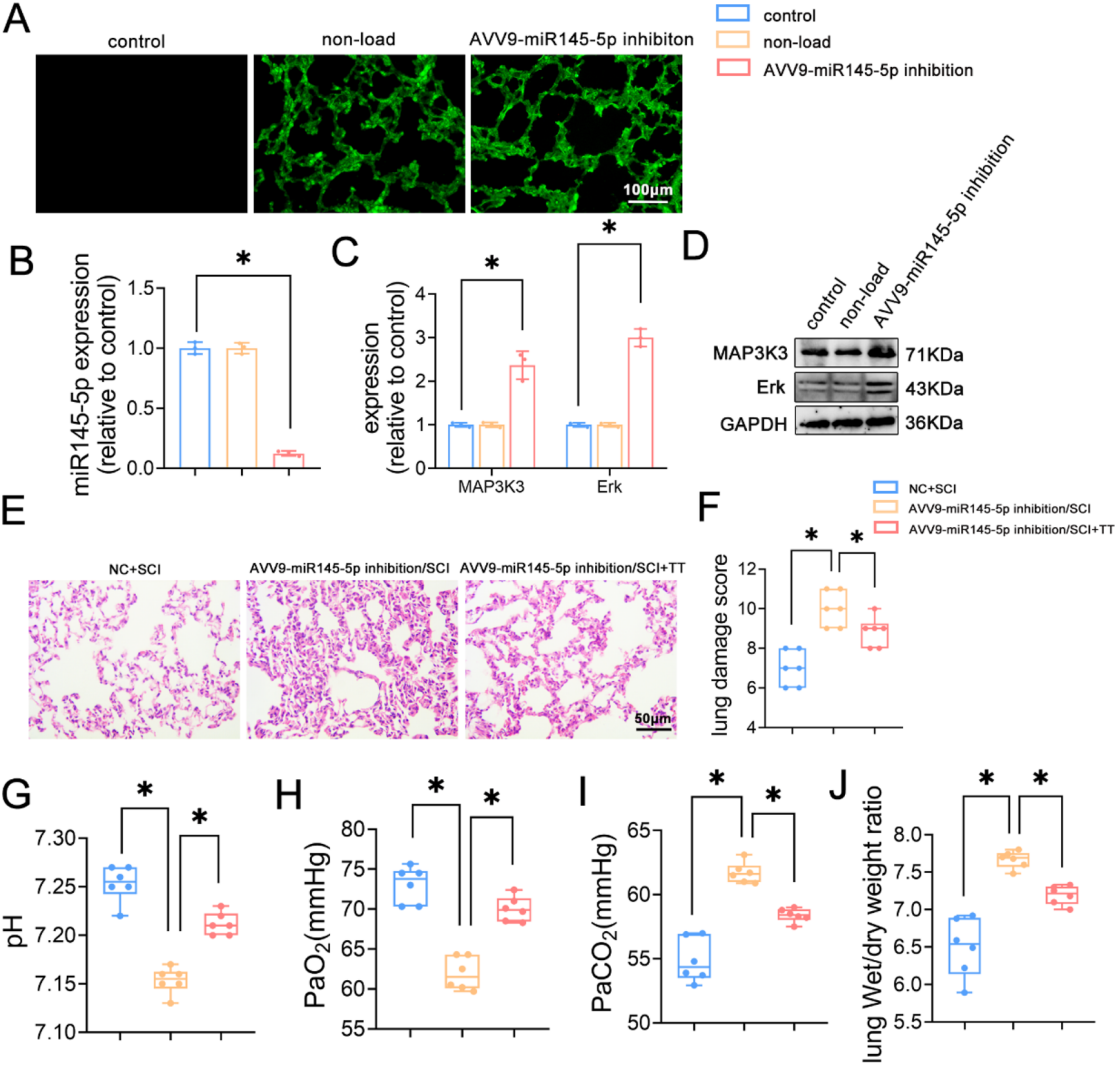
Effects of knockdown of miR-145-5p and treadmill training on lung injury in SCI rats. (**A**) EGFP fluorescent labeling detection, scale bar = 100 µm. (**B**) Relative expression levels of miR145-5p. (**C**) Relative protein expression levels of MAP3K3, Erk. (**D**) Western blotting detection of MAP3K3, Erk expression. (**E**) HE staining results of lung tissues, scale bar = 50 µm. (**F**) Lung pathology score results, n = 6. (**G**) pH level, n = 6. (**H**) PaO_2_ level, n = 6. (**I**) PaCO_2_ level, n = 6. (**J**) Lung wet/dry weight ratio, n = 6. *P < 0.05 as determined by one-way ANOVA (Tukey’s multiple comparison test).

To validate miR145-5p knockdown’s effects, we constructed AAV containing the miR145-5p knockdown plasmid, nebulizing them into rat lung tissues using the same method as overexpression. Examination under fluorescence microscopy revealed the presence of fluorescence in lung tissues of both the non-loaded virus group and the knockdown group, while no such fluorescence was observed in the saline control group (Fig. 7A).

To confirm successful miR145-5p knockdown and its impact on the MAPK signaling pathway, miR145-5p expression and the MAPK signaling pathway were evaluated through qRT-PCR and Western blotting (Fig. 7B–D). In comparison to the control group, the non-loaded group displayed no significant alterations in miR145-5p, MAP3K3, and Erk protein expression levels. In contrast, the knockdown group exhibited significantly reduced miR145-5p expression and notably elevated MAP3K3 and Erk protein expression levels (*P* < 0.05).

The rats underwent a 14-day transfection process involving the introduction of an empty carrier virus and AAV that suppressed miR145-5p expression. This protocol was employed to establish the SCI model. Subsequently, the rats were subjected to treadmill training, both with and without this training intervention. Lung function evaluation for the SCI rats was performed on the 7th day, involving measurement of PaO_2_, PaCO_2_, and pH in the abdominal aorta via arterial blood gas analysis (Fig. 7G–I). Outcomes indicated significantly lower pH and PaO_2_ levels, along with higher PaCO_2_ levels in the AVV9-miR145-5p inhibition/SCI group compared to the NC+SCI group (*p* < 0.05). Conversely, the AVV9-miR145-5p inhibition/SCI+TT group exhibited significantly higher pH and PaO_2_ levels and significantly lower PaCO_2_ levels compared to the AVV9-miR145-5p inhibition/SCI group (*p* < 0.05). Furthermore, an assessment of pulmonary edema through wet/dry weight ratio measurement was performed (Fig. 7J). The results revealed significantly higher wet/dry weight ratios in the AVV9-miR145-5p inhibition/SCI group in contrast to the NC+SCI group and significantly lower ratios in the AVV9-miR145-5p inhibition/SCI+TT group compared to the AVV9-miR145-5p inhibition/SCI group (*p* < 0.05). Moreover, lung tissue changes were evaluated through HE staining, accompanied by pathological scoring (Fig. 7E–F). Inflammatory cell infiltration, edema, congestion, and intra-alveolar hemorrhage were more pronounced in the AVV9-miR145-5p inhibition/SCI group. The AVV9-miR145-5p inhibition/SCI group also exhibited significantly higher pathological scores of lung injury compared to the NC+SCI group, while the AVV9-miR145-5p inhibition/SCI+TT group displayed significantly lower scores than the AVV9-miR145-5p inhibition/SCI group (*p* < 0.05).

In summary, the above results indicate that lung histopathological injury increased and lung function decreased in SCI rats following miR145-5p knockdown. However, treadmill training was effective in ameliorating the extent of lung histopathological injury and improving lung function in rats with miR145-5p knockdown.

*Knockdown of miR145-5p activates the MAPK signaling pathway and promotes apoptosis in rat lung tissues after SCI and treadmill training inhibits the MAPK signaling pathway and attenuates apoptosis.*

**Figure 8:**
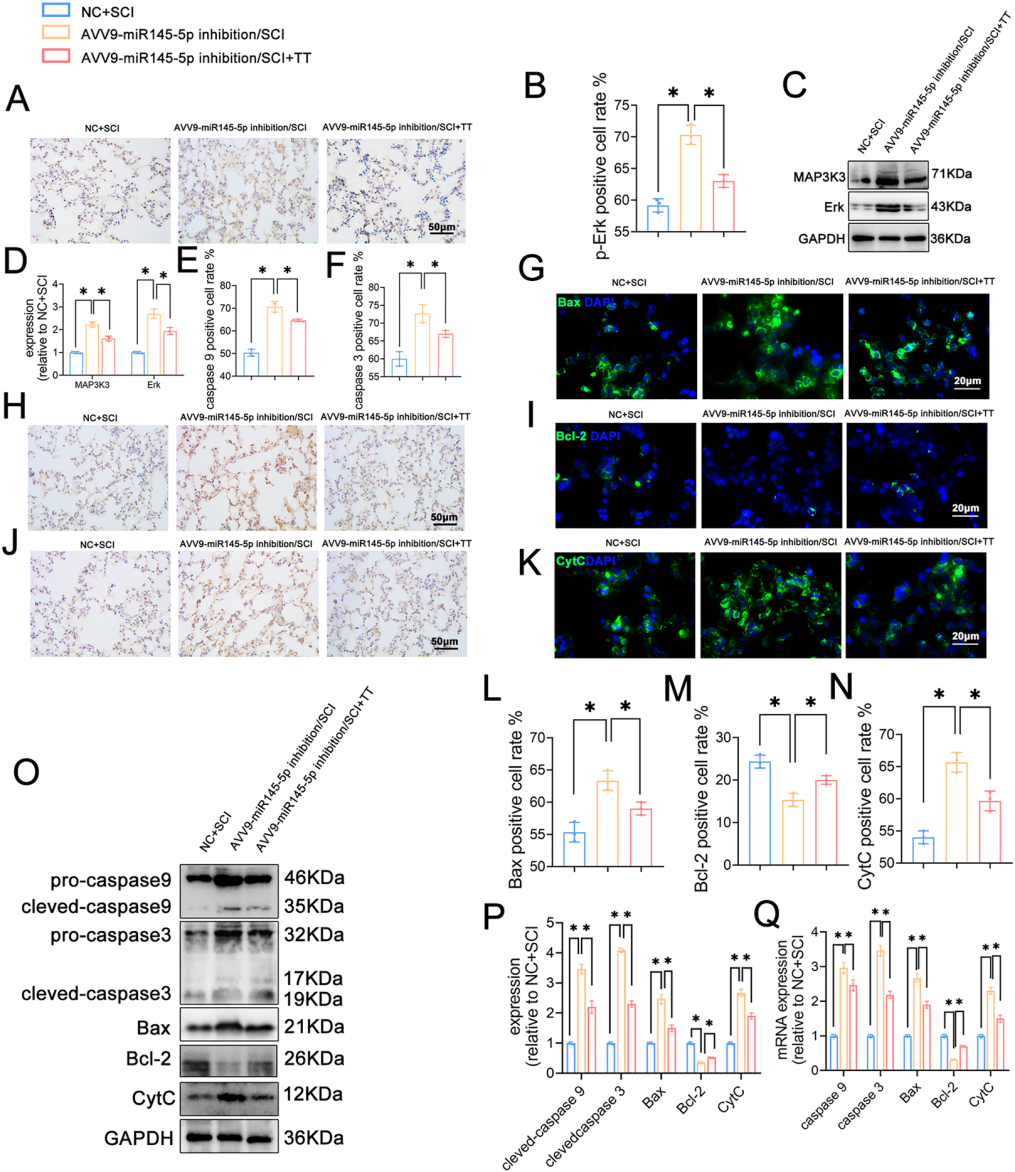
Effects of knockdown of miR145-5p on the MAPK signaling pathway and apoptosis in lung tissues of rats following SCI. (**A**) Immunohistochemical staining of p-Erk, scale bar 50 = µm. (**B**) Rate of p-Erk-positive cells, n = 3. (**C**) Western blotting detection of MAP3K3, Erk expression. (**D**) Relative protein expression levels of MAP3K3, Erk. (**E**) Rate of caspase 9-positive cells, n = 3. (**F**) Rate of caspase 3-positive cells, n = 3. (**G**) Immunofluorescence of Bax staining, scale bar = 20 µm. (**H**) Immunohistochemical staining of caspase 9, scale bar = 20 µm. (**I**) Immunofluorescence staining for Bcl-2, scale bar = 20 µm. (**J**) Immunohistochemical staining for caspase 3, scale bar = 50 µm. (**K**) Immunofluorescence staining for CytC, scale bar = 20 µm. (**L**) Rate of Bax-positive cells, n = 3. (**M**) Rate of Bcl-2-positive cells, n = 3. (**N**) Rate of CytC-positive cells, n = 3. (**O**)Western blotting detection of caspase 9, caspase 3, Bax, Bcl-2, and CytC expression. (**P**) Relative protein expression levels of caspase 9, caspase 3, Bax, Bcl-2 and CytC. (**Q**) Relative expression levels of caspase 9, caspase 3, Bax, Bcl-2, CytC mRNA. *P < 0.05 as determined by one-way ANOVA (Tukey’s multiple comparison test).

Immunohistochemical staining (Fig. 8A,B) indicated a significantly higher expression of p-Erk in the AVV9-miR145-5p inhibition/SCI group compared to the NC+SCI group (*P* < 0.05). Moreover, this expression was lower in the AVV9-miR145-5p inhibition/SCI+TT group in contrast to the AVV9-miR145-5p inhibition/SCI group (*P* < 0.05). Similarly, Western blotting (Fig. 8C,D) demonstrated significantly higher MAP3K3 and Erk protein levels in the AVV9-miR145-5p inhibition/SCI group than in the NC+SCI group (*P* < 0.05). Conversely, compared to the AVV9-miR145-5p inhibition/SCI group, the AVV9-miR145-5p inhibition/SCI+TT group displayed lower MAP3K3 and Erk protein expression levels (*P* < 0.05).

We further examined apoptosis in lung tissues. Immunohistochemical staining results (Fig. 8E,F,H,J) demonstrated significantly increased expression of caspase 9 and caspase 3 in the AVV9-miR145-5p inhibition/SCI group compared to the NC+SCI group (*P* < 0.05). Conversely, the AVV9-miR145-5p inhibition/SCI+TT group exhibited reduced expression in comparison to the AVV9-miR145-5p inhibition/SCI group (*P* < 0.05). Immunofluorescence staining (Fig. 8G,I,K–N) further supported these findings, revealing increased expression levels of Bax and CytC, and decreased expression levels of Bcl-2 in the AVV9-miR145-5p inhibition/SCI group compared to the NC+SCI group (*P* < 0.05). Additionally, when compared to the AVV9-miR145-5p inhibition/SCI group, the AVV9-miR145-5p inhibition/SCI+TT group exhibited decreased expression levels of Bax and CytC, and increased levels of Bcl-2 (*P* < 0.05). Furthermore, Western blotting and qRT-PCR (Fig. 8O–Q) indicated decreased Bcl-2 protein and mRNA expression levels in the AVV9-miR145-5p inhibition/SCI group relative to the NC+SCI group, along with significantly higher expression levels of caspase 9, caspase 3, Bax, and CytC protein and mRNA (*P* < 0.05). In comparison, the AVV9-miR145-5p inhibition/SCI+TT group displayed significantly higher Bcl-2 mRNA and protein expression levels and notably lower caspase 9, caspase 3, Bax, and CytC mRNA and protein expression levels than the AVV9-miR145-5p inhibition/SCI group (*P* < 0.05).

In summary, the results reveal that miR145-5p knockdown exacerbated activation of the MAPK signaling pathway and increased apoptosis in lung tissues of SCI rats. On the other hand, treadmill training could inhibit the MAPK signaling pathway and mitigate apoptosis in lung tissues of rats post-SCI with miR145-5p knockdown.

## Discussion

The findings of this study indicate that treadmill training can potentially enhance lung injury recovery in rats with SCI. The underlying mechanism appears to involve the reduction of lung cell apoptosis by targeting the MAP3K3 to regulate the MAPK/Erk signaling pathway through a specific molecule, miR145-5p. This study not only highlights the potential of treadmill training as a therapeutic approach for SCI-related lung injury but also provides novel insights into the rehabilitation of such injuries.

Previous research has indicated that SCI can lead to respiratory complications, potentially linked to diminished lung capacity, retention of secretions, and autonomic dysfunction^33^. Indeed, the extent of respiratory dysfunction varies based on the location of the SCI. Cervical and upper thoracic injuries often result in impaired respiratory muscle function due to loss of innervation. Interestingly, even segments with functional respiratory muscles, like the T10 segmental spinal cord, typically exhibit pathological changes in lung tissue after SCI, such as edema, hemorrhage, and inflammation. These changes could be attributed to various factors like apoptosis, inflammatory response, and autophagy pathways^6,9,10^. It is now understood that neutrophil and monocyte migration to lung tissues hours after SCI contributes to lung tissues damage, while mitochondrial dysfunction and alveolar cell apoptosis have been observed as well^8,34^.

Exercise training is a common rehabilitative strategy for SCI patients, with evidence suggesting its ability to improve lung function after injury^35^. In a clinical trial, Duan et al.^36^ observed that modified Total Body Recumbent Stepper (TBRS) training effectively improved exercise capacity, alleviated dyspnea symptoms, and reduced acute exacerbations in patients with chronic obstructive pulmonary disease (COPD). Moreover, exoskeleton-assisted walking training demonstrated the ability to enhance lung function parameters in SCI patients, while weight-supported treadmill training was shown to boost cardiopulmonary function in the same population^37,38^. Exercise training has also displayed beneficial outcomes in addressing lung dysfunction stemming from various respiratory diseases in animal studies. For instance, Qin et al.^39^ discovered that eight weeks of treadmill exercise prior to PM2.5 exposure mitigated lung injury in aging rats, possibly through the inhibition of the TLR4/NF-κB signaling pathway. Additionally, aerobic exercise training alleviated airway obstruction, lowered respiratory muscle strength, reduced bronchial mucosal damage, mitigated ultrastructural harm to lung tissue, and suppressed inflammatory responses in rats with PM2.5-induced lung injury, with these improvements correlated with diminished activation of p38 and MAPK pathways^40^. However, the precise mechanisms through which exercise ameliorates lung injury in the context of SCI remain to be elucidated.

Recent studies have unveiled the pivotal role of apoptosis in the development of lung injuries. For instance, Reduning was found to reduce sepsis-induced lung injury through AKT1 targeting to suppress apoptosis in lung microvascular endothelial cells^41^. Artesunate, in mice with intestinal ischemia/reperfusion-induced lung injury, was found to alleviate apoptosis via upregulation of AKT and HO-1 signaling pathways^42^. Moreover, AMPK exhibited the capacity to ameliorate pneumonia-induced lung injury by mitigating alveolar epithelial cell apoptosis^43^. Apoptosis might also be a central mechanism through which exercise training confers benefits against lung injury. For instance, swimming exercise was shown to mitigate lung-related lesions in type I diabetic mice through the regulation of apoptosis^44^. Consistently, treadmill training has been observed to counteract lung tissue damage during PM exposure in mice by eliminating damaged mitochondria and reducing apoptosis; it similarly ameliorated COPD-induced lung injury through apoptosis reduction and sirt1 activation^14,45^. In the present study, we found that treadmill training could decrease the expression levels of pro-apoptotic factors, including Bax, CytC, caspase 3, and caspase 9, alongside an increase in the expression levels of the anti-apoptotic factor Bcl-2. Mechanistically, Bax competitively bound to the apoptosis-inhibiting protein Bcl-2, releasing Bax/Bak from inhibition. This resulted in altered mitochondrial membrane permeability, releasing CytC and activating the caspase cascade, ultimately triggering apoptosis. CytC further bound to apoptosis-associated factor 1 (Apaf-1), recruiting caspase 9 precursors within the cytoplasm, with activated caspase 9 subsequently inducing caspase 3-mediated apoptosis^46^. Taken together, these findings suggest that treadmill training potentially mitigates lung injury by attenuating lung cell apoptosis in SCI rats.

To uncover the potential mechanism underlying the improvement of lung cell apoptosis in rats with SCI through treadmill training, we quantified the expression of the MAPK cascade in lung tissues post-SCI. Our investigation revealed an elevation in the expression levels of MAP3K3 and p-Erk in lung tissues after SCI, whereas treadmill training could downregulate the expression of both MAP3K3 and p-Erk. MAP3K3 holds significance as an essential upstream controller of the MAPK signaling pathway, playing a pivotal role in its regulation. The MAPK/Erk pathway’s involvement spans various facets of cellular growth and development, including apoptosis, proliferation, differentiation, migration, and senescence. It has been demonstrated that the MAPK/Erk signaling pathway is implicated in the progression of diverse lung ailments. For instance, irigenin was observed to mitigate LPS-induced lung injury through the inhibition of lung cell apoptosis by restraining the MAPK/Erk signaling pathway^47^. In our study, treadmill training exhibited the ability to suppress the expression of MAP3K3 and p-Erk, resulting in reduced expression of pro-apoptotic factors such as Bax, CytC, caspase 3, and caspase 9, coupled with upregulated expression of the anti-apoptotic factor Bcl-2 within lung tissues of SCI rats. From this, we formulated a hypothesis that treadmill training’s potential to alleviate lung injury in SCI rats might stem from its capability to mitigate lung cell apoptosis through the inhibition of the MAPK/Erk signaling pathway.

To further explore the mechanism underlying treadmill training’s therapeutic effect following SCI-induced lung injury, we assessed the potential involvement of miR145-5p in the pathogenesis and progression of lung injury post-SCI. Our hypothesis was further substantiated by bioinformatics databases, predicting MAP3K3 as the target gene of miR145-5p, a conjecture that was subsequently confirmed via dual luciferase reporter gene assay. Given that MAP3K3 operates upstream in the MAPK signaling pathway, it is highly conceivable that miR145-5p might govern the MAPK pathway by targeting MAP3K3. This occurs through the direct binding of miR145-5p to MAP3k3 mRNA, resulting in the downregulation of MAP3k3 expression. To explore miR145-5p’s regulatory role in the context of SCI-induced lung injury, we conducted experiments involving the overexpression or knockdown of miR145-5p in rat lungs. In light of previous findings indicating that lung cell apoptosis peaked at 48 and 72 h post-SCI^10^. While the differences in apoptotic factor expression between the SCI group and the SCI+TT group were significant on days 3 and 7 in our study, the brevity of the 3-day treadmill training period made it less suitable for assessing its impact on lung injury. Consequently, we opted to assess lung function and lung cell apoptosis in SCI rats on day 7. Our findings indicated that the overexpression of miR145-5p led to decreased MAP3K3 expression, inhibited the MAPK signaling pathway, and ultimately mitigated lung cell apoptosis in SCI rats. Remarkably, overexpressing miR145-5p appeared to be more effective in reducing lung dysfunction compared to treadmill training alone. Conversely, miR145-5p knockdown led to upregulation of MAP3K3 expression, activating the MAPK signaling pathway, and exacerbated lung cell apoptosis in SCI rats. Intriguingly, treadmill training was found to promote the expression of miR145-5p, which in turn led to the amelioration of lung cell apoptosis in rats with miR145-5p knockdown. This protective role of miR145-5p against lung injury has been established in prior research, exemplified by its attenuation of LPS-induced lung injury through the inactivation of the TGF-β1/Smad signaling pathway via targeting ETS2^48^. Interestingly, miR-145 demonstrated amelioration of sepsis-induced lung injury through the inhibition of TGFBR2 signaling in another study^49^. Similarly, in a mouse model of LPS-induced lung injury, overexpression of miR145-5p led to a reduction in pro-inflammatory cytokine expression and mitigated lung tissue damage^50^. The present study findings corroborate that treadmill training’s influence on the MAPK/Erk signaling pathway via targeting MAP3K3 through miR145-5p results in reduced lung cell apoptosis, ultimately offering a protective effect on lung tissues in SCI rats.

To conclude, our research provides compelling evidence that treadmill training has the potential to diminish lung cell apoptosis and enhance lung function in rats with SCI. Additionally, we have elucidated the potential mechanism underlying treadmill training’s effects, which could be mediated by the interaction between miR145-5p and MAP3K3, leading to the modulation of the MAPK/Erk signaling pathway and the consequent inhibition of lung cell apoptosis. Nevertheless, it is important to acknowledge our study’s limitations, including the absence of comprehensive confirmation through in vitro cellular-level investigations. Indeed, further research is paramount to expand our understanding of the therapeutic impact of treadmill training on SCI-induced lung injuries.

## Supplementary Information

Supplementary Figures.

## Data Availability

All data generated or analyzed during this study are included in this published article. All experiments were conducted in accordance with relevant guidelines and regulations. The study is reported in accordance with ARRIVE guidelines.
