# Author Correction: Treadmill training improves lung function and inhibits alveolar cell apoptosis in spinal cord injured rats

**DOI:** 10.1038/s41598-024-84228-z

**Published:** 2025-01-16

**Authors:** Xianbin Wang, Yingxue Fu, Xianglian Yang, Yan Chen, Ni Zeng, Shouxing Hu, Shuai Ouyang, Xiao Pan, Shuang Wu

**Affiliations:** 1 Affiliated Hospital of Guizhou Medical University, 28 Guiyi Street, Yunyan District, Guiyang, Guizhou China; 2 Guizhou Medical University, 9 Beijing Street, Yunyan District, Guiyang, Guizhou China

Correction to: *Scientific Reports* 10.1038/s41598-024-59662-8, published online 27 April 2024

In the original version of this Article, Figs. 4 panel A and 8 panel A were duplicated, and Figs. 6P (Western blot, pro-caspase3) and 8O (Western blot, Bcl-2) were incorrectly assigned due to group misplacement.

The original Figs. 4 and 8 and their accompanying legends appear below.

**Fig. 4:**
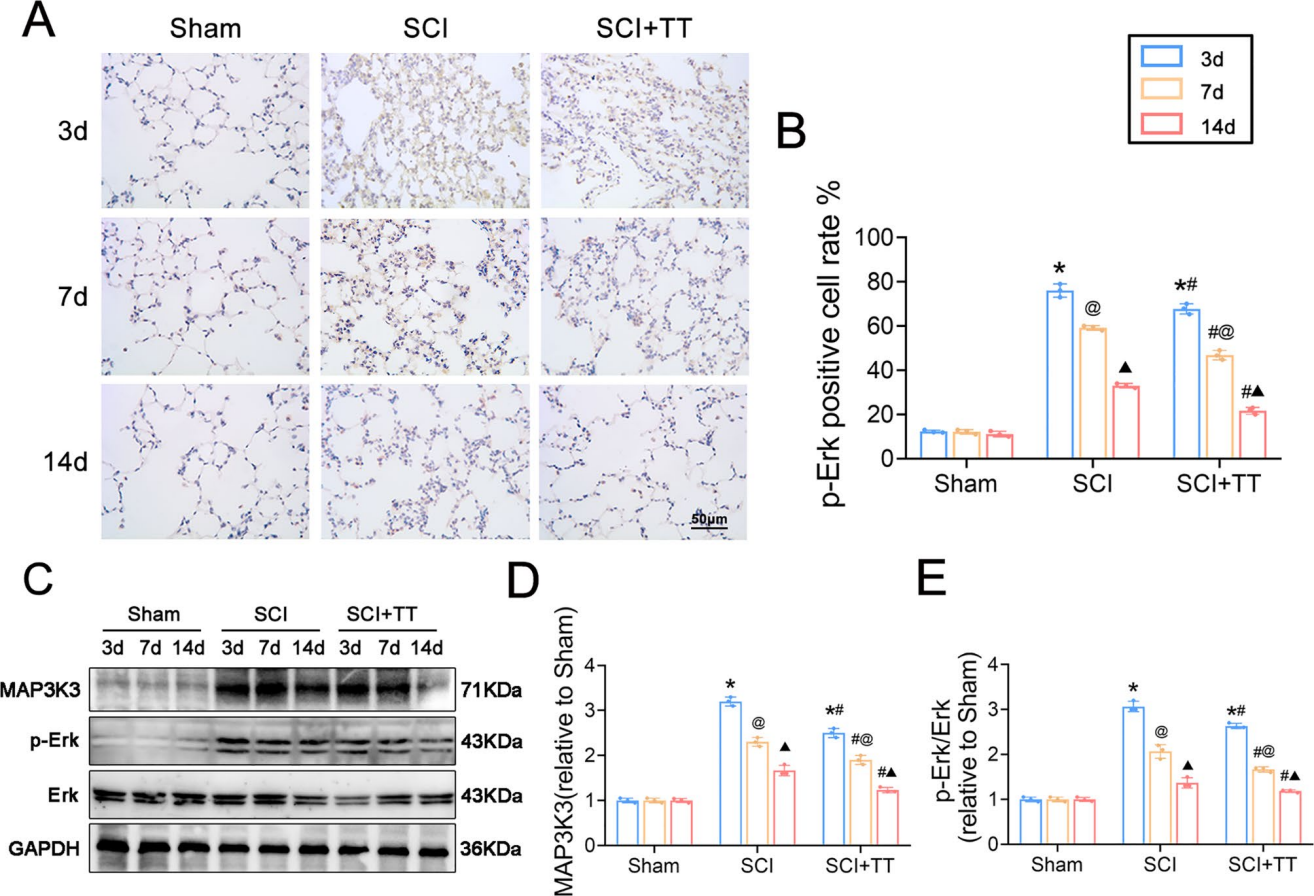
Effects of treadmill training on the MAPK signalling pathway in lung tissue of rats with SCI. (**A**) Immunohistochemical staining of p-Erk, scale bar = 50 µm. (**B**) Rate of p-Erk positive cells, n = 3. (**C**) Western blotting detection of MAP3K3 and p-Erk protein expression. (**D**) MAP3K3 protein relative expression levels. (**E**) Relative protein expression levels of p-Erk. *P < 0.05, compared with the Sham group at the same time point; #*P* < 0.05, compared with the SCI group at the same time point; @*P* < 0.05, compared with the same group on day 3; ▲ *P* < 0.05, compared with the same group on day 7. *P* < 0.05 was determined by two-way ANOVA (Bonferroni’s multiple comparison test).

**Fig. 8:**
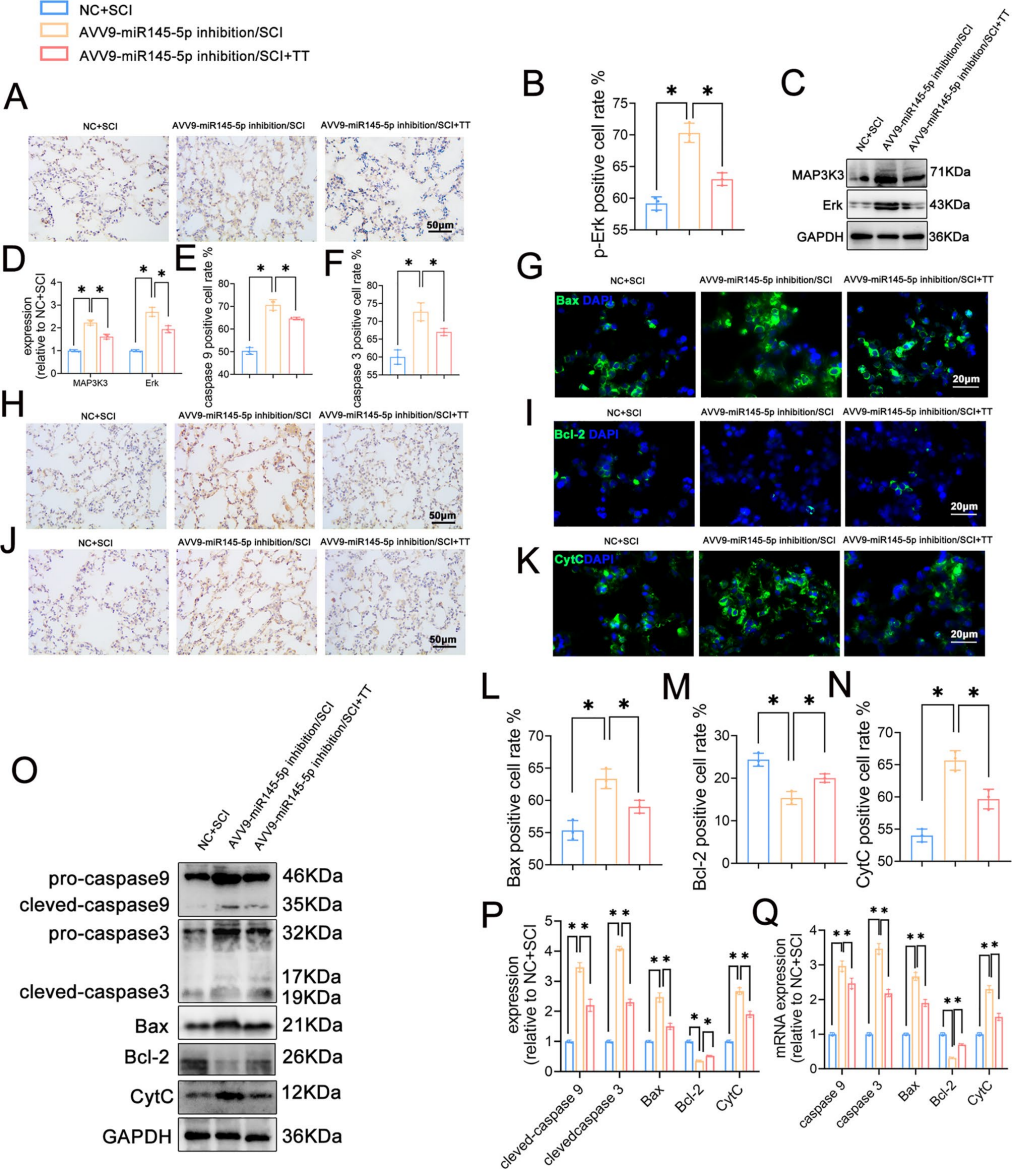
Effects of knockdown of miR145-5p on the MAPK signaling pathway and apoptosis in lung tissues of rats following SCI. (**A**) Immunohistochemical staining of p-Erk, scale bar 50 = µm. (**B**) Rate of p-Erk-positive cells, n = 3. (**C**) Western blotting detection of MAP3K3, Erk expression. (**D**) Relative protein expression levels of MAP3K3, Erk. (**E**) Rate of caspase 9-positive cells, n = 3. (**F**) Rate of caspase 3-positive cells, n = 3. (**G**) Immunofluorescence of Bax staining, scale bar = 20 µm. (**H**) Immunohistochemical staining of caspase 9, scale bar = 20 µm. (**I**) Immunofluorescence staining for Bcl-2, scale bar = 20 µm. (**J**) Immunohistochemical staining for caspase 3, scale bar = 50 µm. (**K**) Immunofluorescence staining for CytC, scale bar = 20 µm. (**L**) Rate of Bax-positive cells, n = 3. (**M**) Rate of Bcl-2-positive cells, n = 3. (**N**) Rate of CytC-positive cells, n = 3. (**O**)Western blotting detection of caspase 9, caspase 3, Bax, Bcl-2, and CytC expression. (**P**) Relative protein expression levels of caspase 9, caspase 3, Bax, Bcl-2 and CytC. (**Q**) Relative expression levels of caspase 9, caspase 3, Bax, Bcl-2, CytC mRNA. *P < 0.05 as determined by one-way ANOVA (Tukey’s multiple comparison test).

The original Article has been corrected.

