# Supplementary material for: Treadmill training improves lung function and inhibits alveolar cell apoptosis in spinal cord injured rats
Source: Sci Rep. 2024 Apr 27;14:9723. doi: 10.1038/s41598-024-59662-8 (PMC11055912; doi:10.1038/s41598-024-59662-8)

**Original images of western blotting in Fig.3**


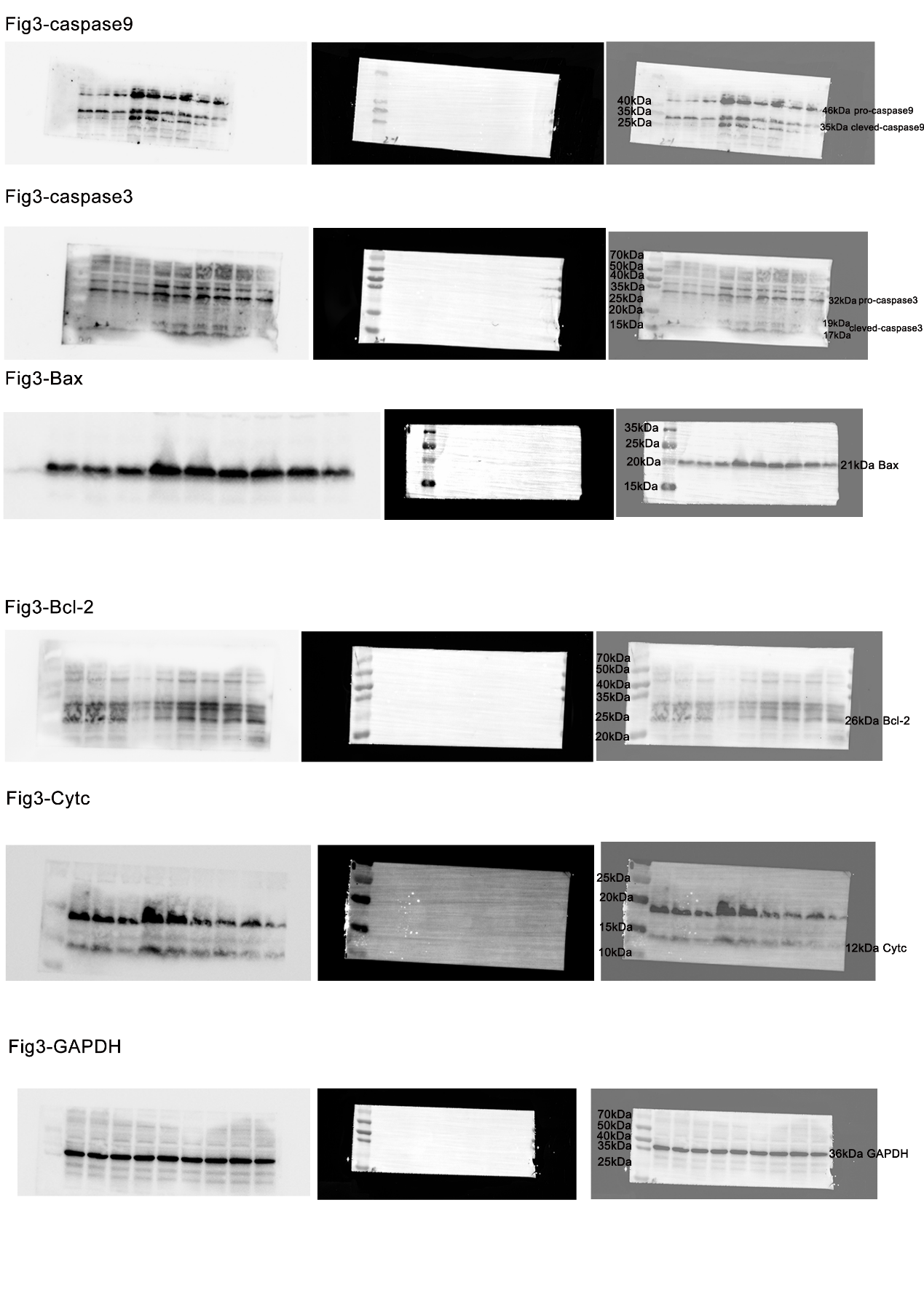


**Original images of western blotting in Fig.4**


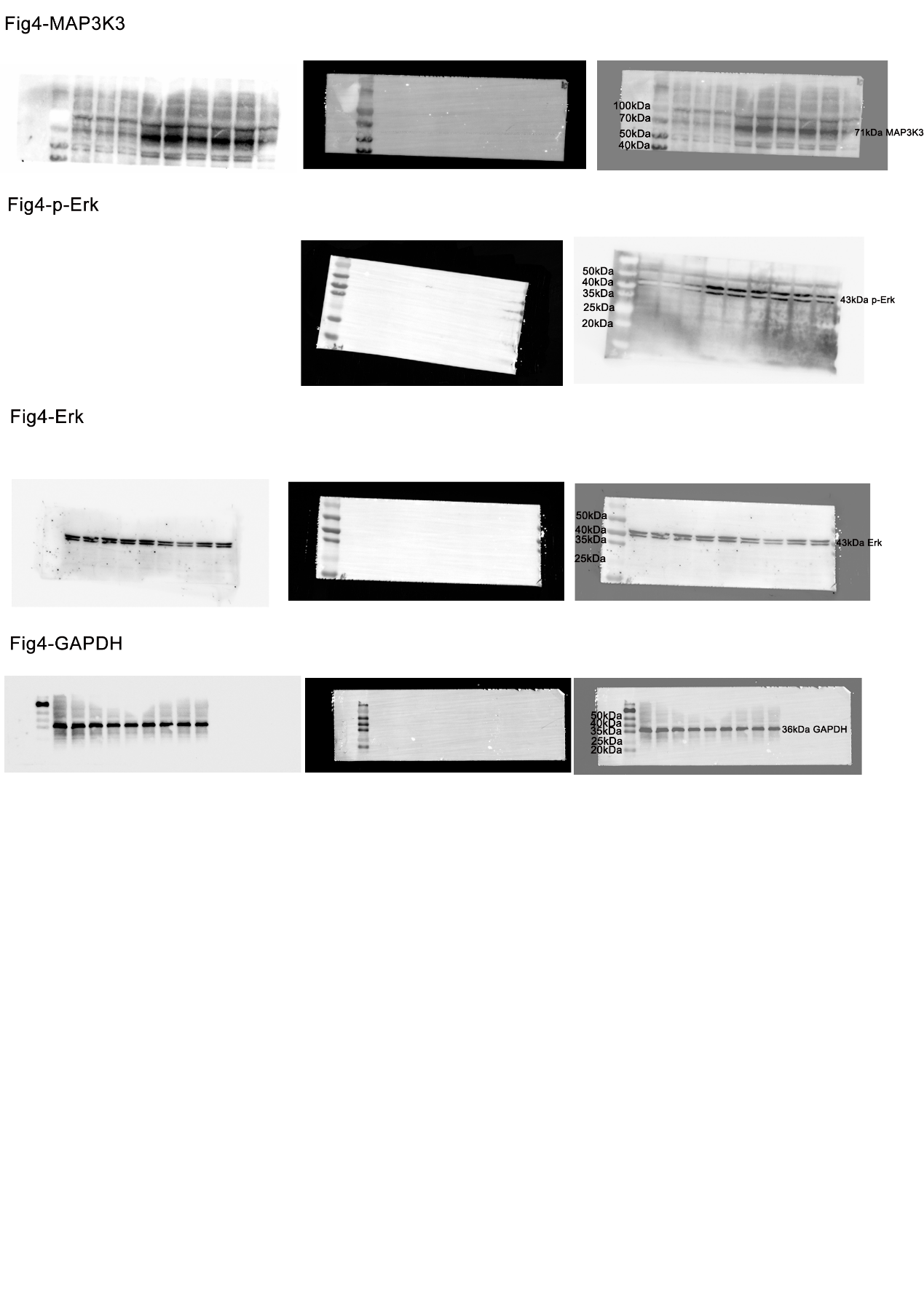


**Original images of western blotting in Fig.5**


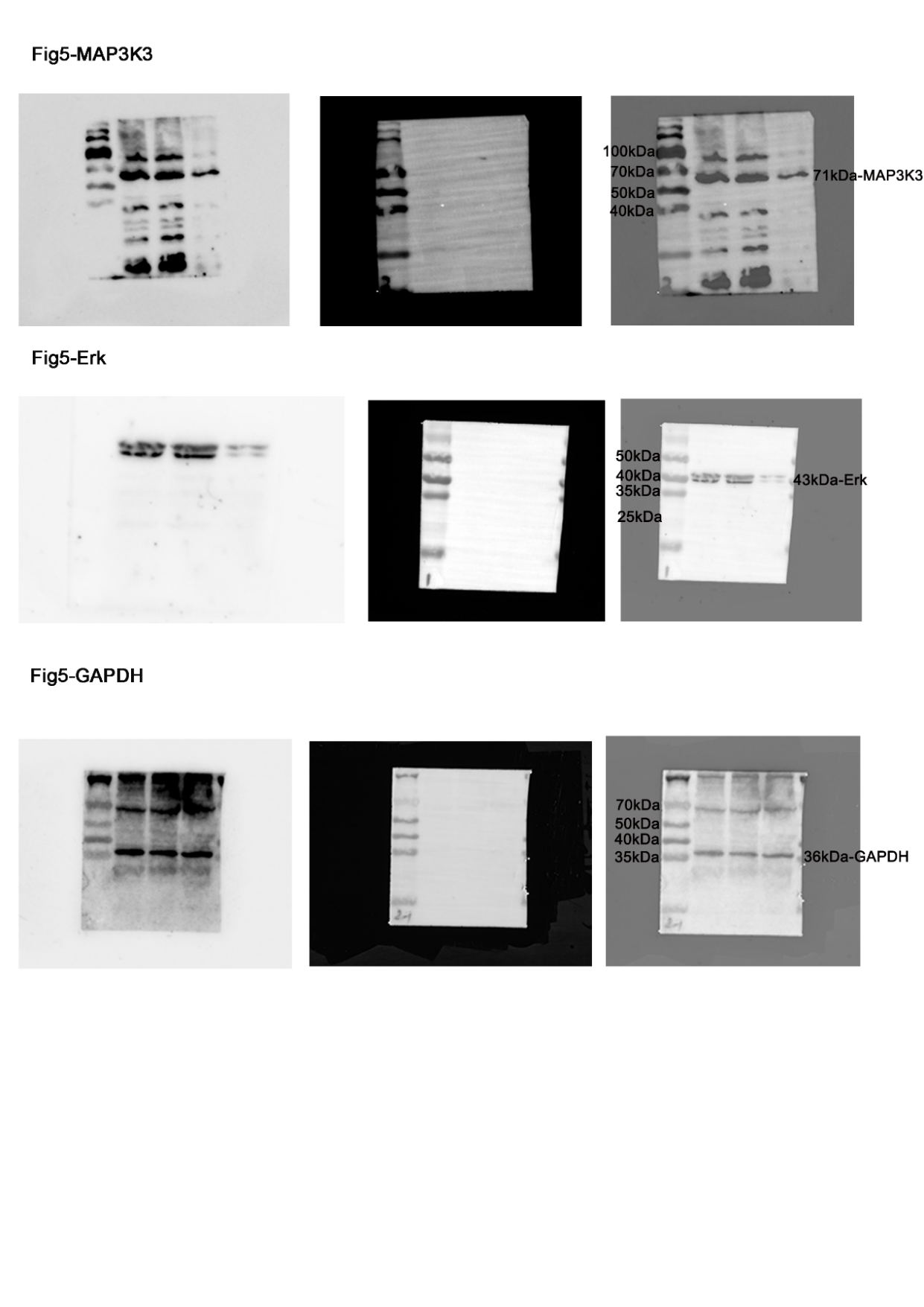


**Original images of western blotting in Fig.6**


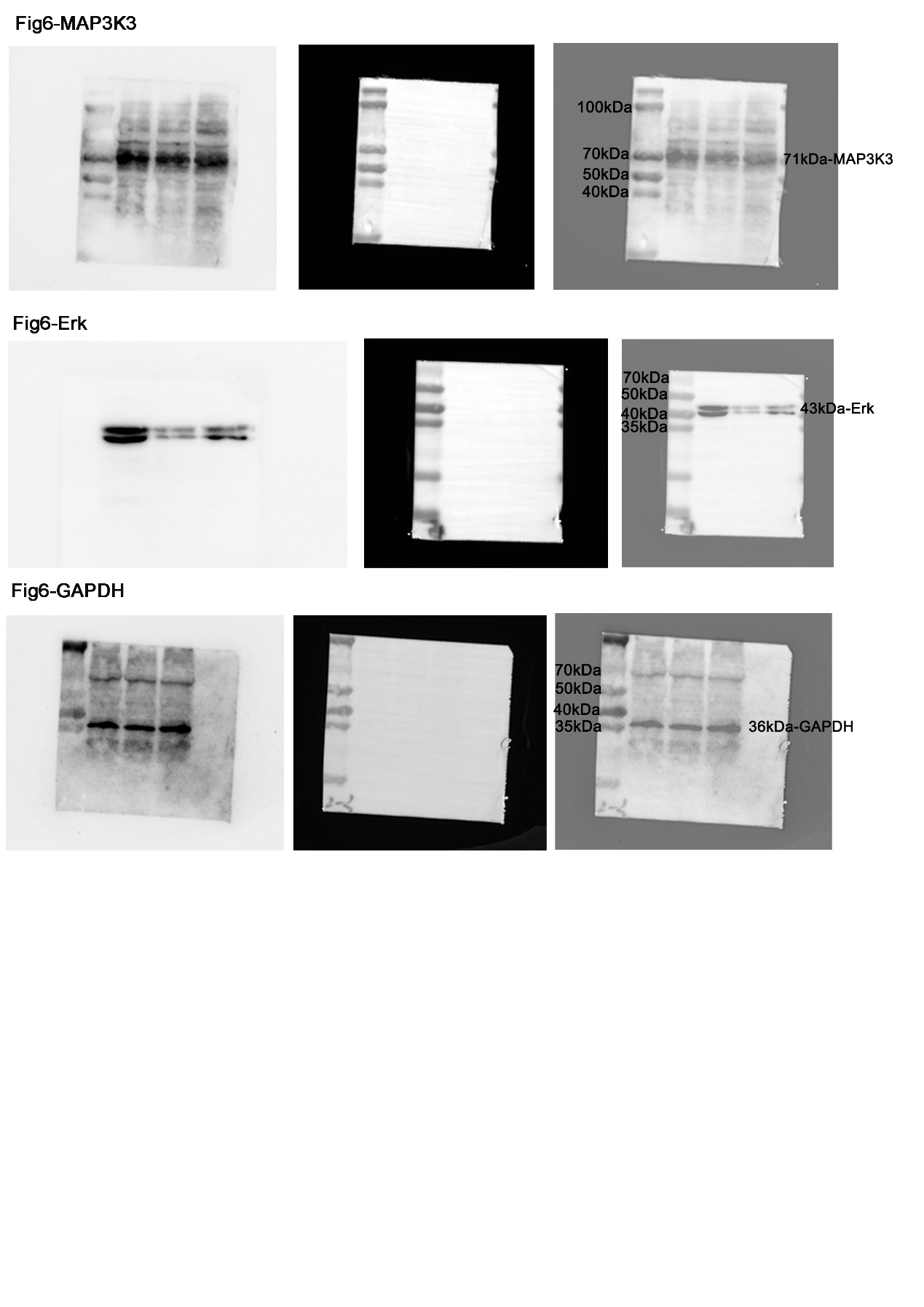


**Original images of western blotting in Fig.6**


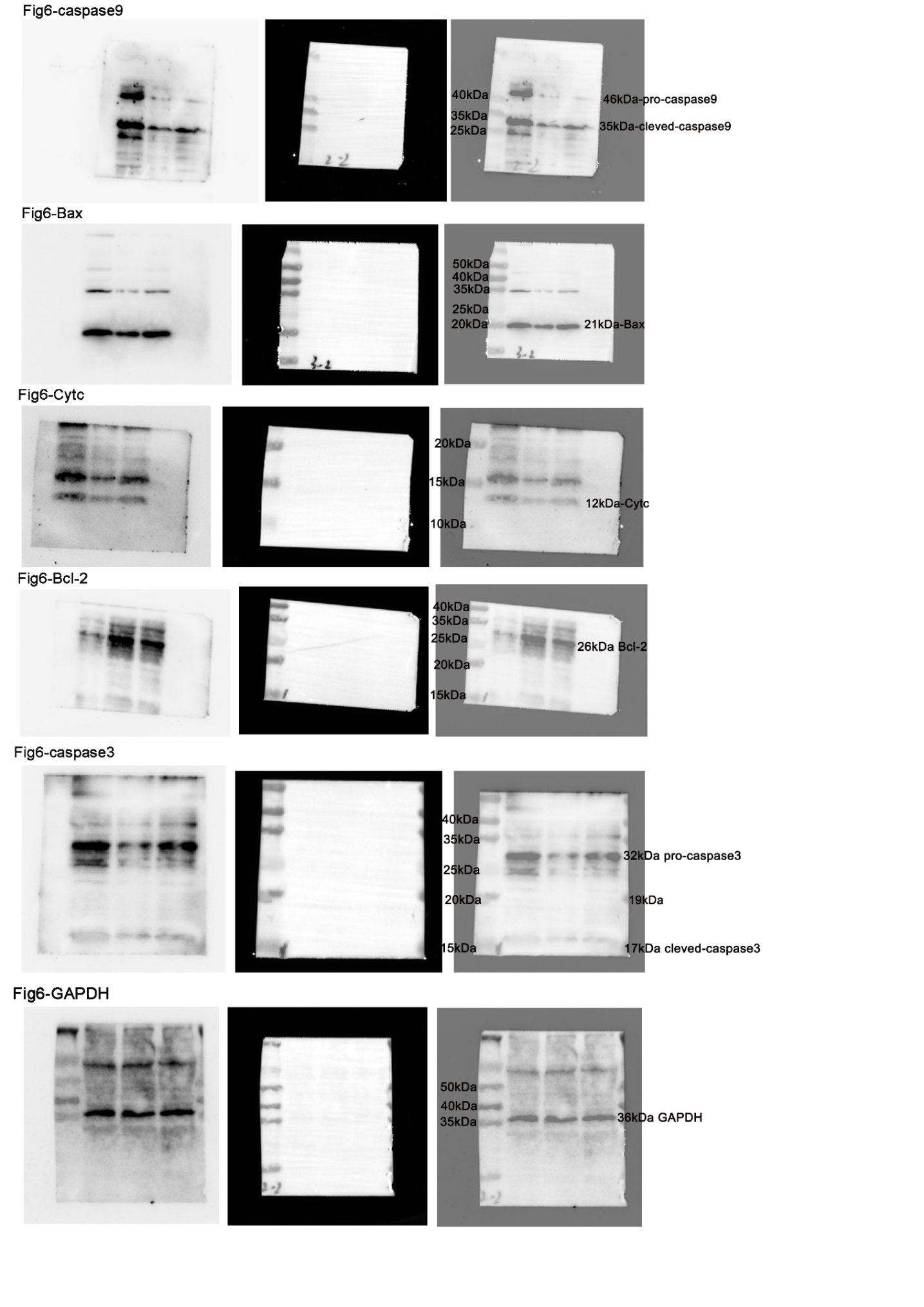


**Original images of western blotting in Fig.7**


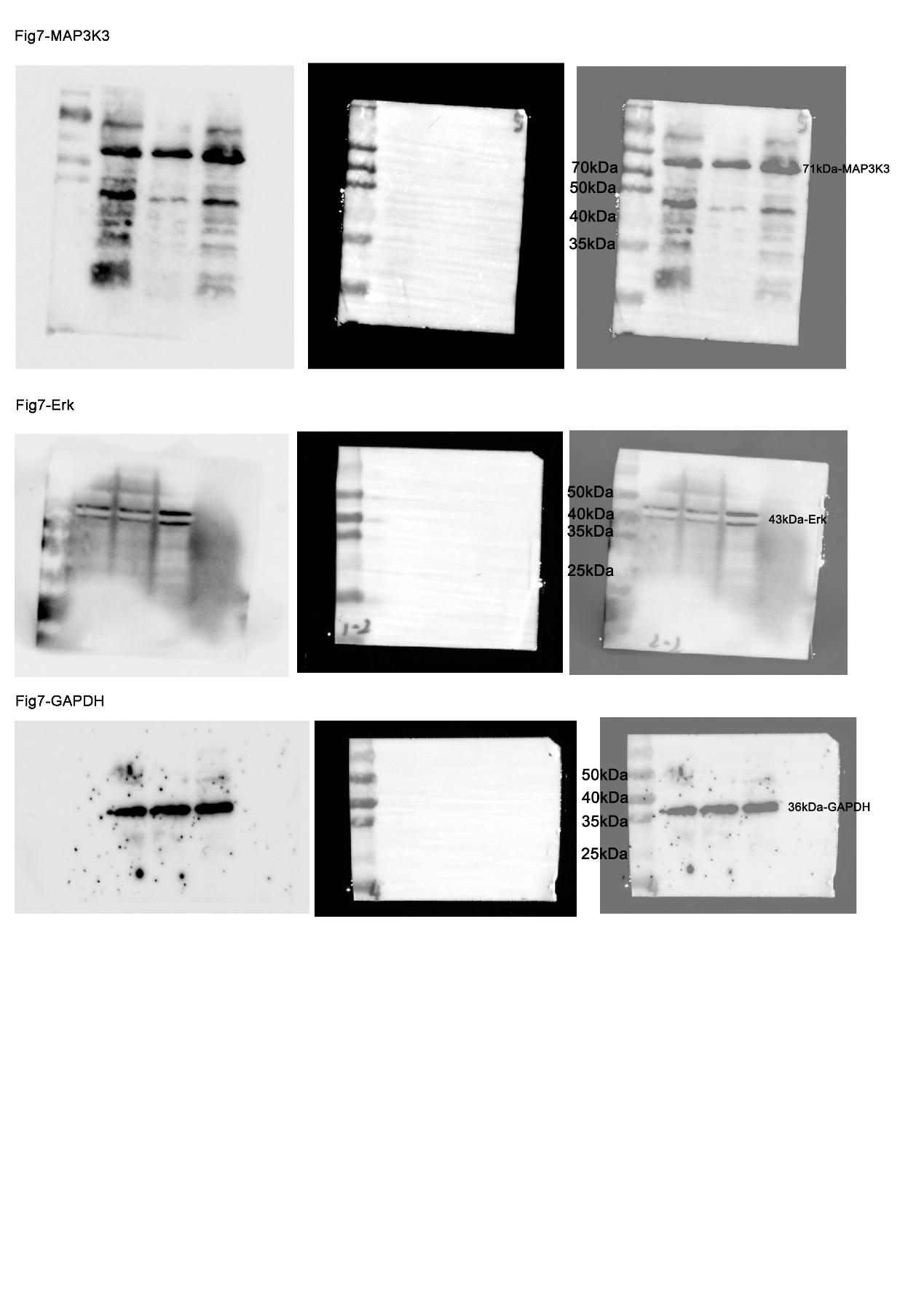


**Original images of western blotting in Fig.8**


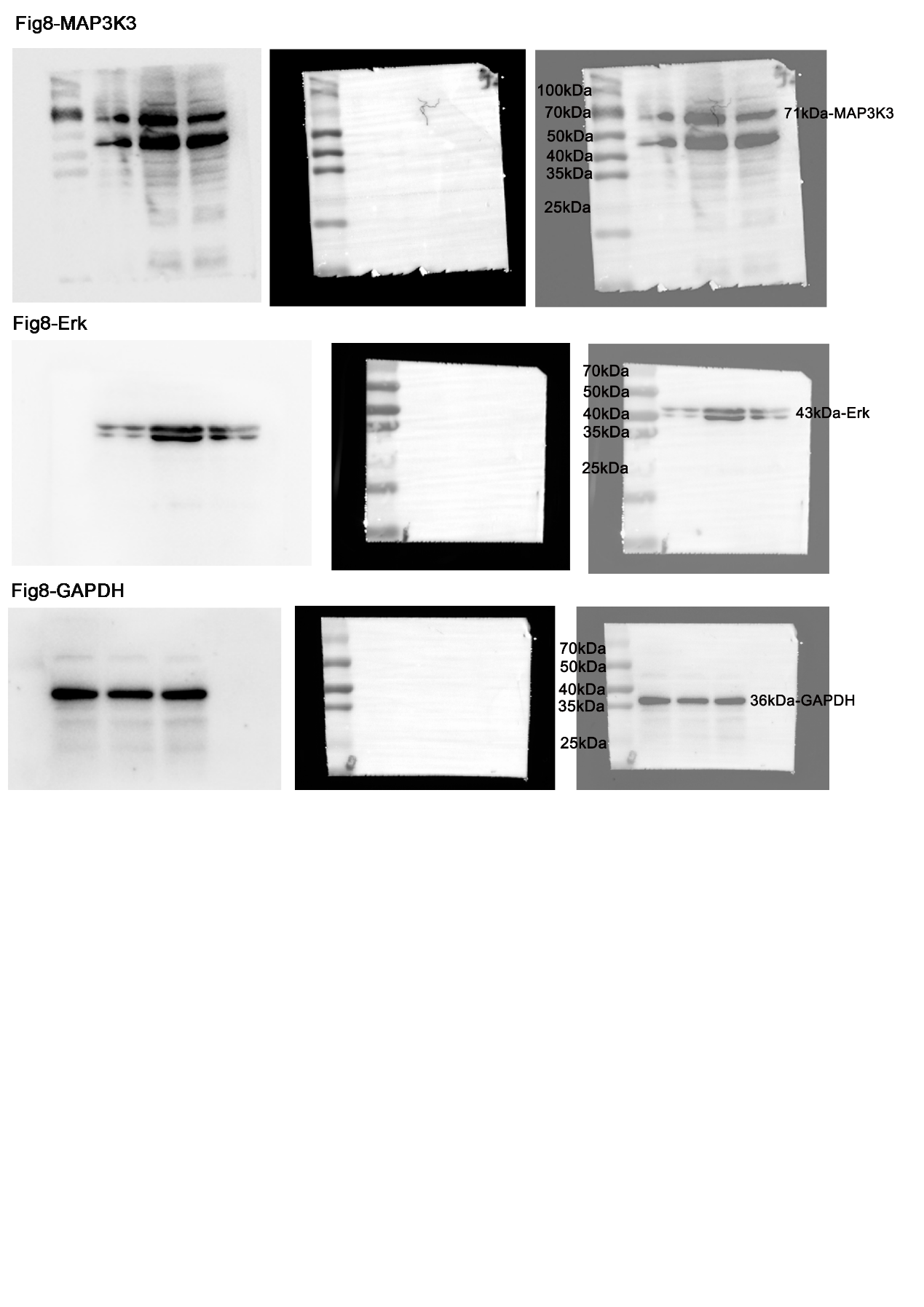


**Original images of western blotting in Fig.8**


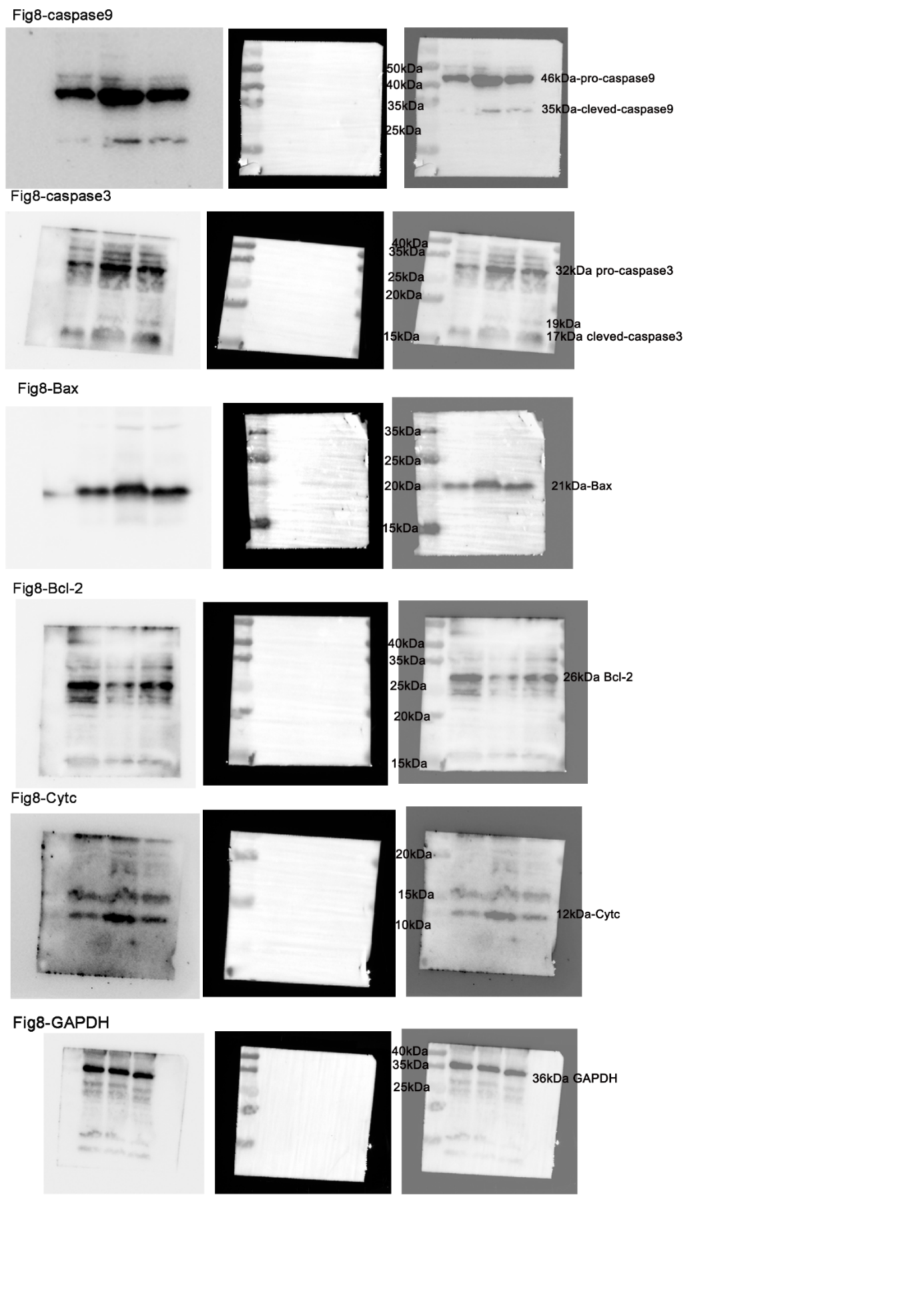

Supplement: Supplementary file 1 — Supplementary Figures. [file 41598_2024_59662_MOESM1_ESM.docx]
